# *Borrelia burgdorferi* and *Borrelia miyamotoi* in Atlantic Canadian wildlife

**DOI:** 10.1371/journal.pone.0262229

**Published:** 2022-01-21

**Authors:** Christopher B. Zinck, Vett K. Lloyd

**Affiliations:** 1 Western College of Veterinary Medicine, University of Saskatchewan, Saskatchewan, Canada; 2 Department of Biology, Mount Allison University, Sackville, New Brunswick, Canada; University of Kentucky College of Medicine, UNITED STATES

## Abstract

*Borrelia burgdorferi* and *Borrelia miyamotoi* are tick-vectored zoonotic pathogens maintained in wildlife species. Tick populations are establishing in new areas globally in response to climate change and other factors. New Brunswick is a Canadian maritime province at the advancing front of tick population establishment and has seen increasing numbers of ticks carrying *B*. *burgdorferi*, and more recently *B*. *miyamotoi*. Further, it is part of a region of Atlantic Canada with wildlife species composition differing from much of continental North America and little information exists as to the presence and frequency of infection of *Borrelia spp*. in wildlife in this region. We used a citizen science approach to collect a wide range of animals including migratory birds, medium-sized mammals, and small mammals. In total we tested 339 animals representing 20 species for the presence of *B*. *burgdorferi* and *B*. *miyamotoi*. We have developed new nested PCR primers and a protocol with excellent specificity for detecting both of these *Borrelia* species, both single and double infections, in tissues and organs of various wildlife species. The positive animals were primarily small non-migratory mammals, approximately twice as many were infected with *B*. *burgdorferi* than *B*. *miyamotoi* and one animal was found infected with both. In addition to established reservoir species, the jumping mouse (*Napaeozapus insignis*) was found frequently infected; this species had the highest infection prevalence for both *B*. *burgdorferi* and *B*. *miyamotoi* and has not previously been identified as an important carrier for either *Borrelia* species. Comprehensive testing of tissues found that all instances of *B*. *burgdorferi* infection were limited to one tissue within the host, whereas two of the five *B*. *miyamotoi* infections were diffuse and found in multiple systems. In the one coinfected specimen, two fetuses were also recovered and found infected with *B*. *miyamotoi*. This presumptive transplacental transmission suggests that vertical transmission in mammals is possible. This finding implies that *B*. *miyamotoi* could rapidly spread into wildlife populations, as well as having potential human health implications.

## Introduction

*Borrelia burgdorferi* and *B*. *miyamotoi* are zoonotic spirochete bacteria found throughout the world. *B*. *burgdorferi* is the principal causative agent of Lyme disease in North America, whereas *B*. *miyamotoi* causes *Borrelia miyamotoi* disease, a relapsing fever-like disease not unlike Lyme disease [1]. These two bacteria are vectored by Ixodid ticks, primarily *Ixodes scapularis* and *Ixodes pacificus* in North America [2–5]. Both *Borrelia* species propagate through a series of reciprocal vector and host transmissions. Larval and nymphal ticks acquire the bacteria by feeding on previously tick-infected animals, they maintain the bacteria through molting (transtadial transmission) to then transmit the infection to future hosts, which can thus receive pathogens collected from all prior hosts [5, 6]. In addition, *B*. *miyamotoi* is able to be transmitted transovarially in ticks to allow the production of ticks infected from hatching [7, 8].

*Ixodes* species ticks have been expanding their range globally in response to climate change and natural and anthropomorphic factors [9–12]. New Brunswick, Canada is an Atlantic Canadian province bordered by the Atlantic Ocean and, on land, by Maine, USA, Quebec, Canada, and Nova Scotia, Canada and is on the emerging front of tick population establishment [10]. Endemic regions are regions where *I*. *scapularis* populations are known to be established and both ticks and wildlife are found infected with *B*. *burgdorferi*. It can be challenging to document newly established endemic regions. At the time of this study, New Brunswick had two identified tick endemic regions in the southwest portion of the province, however, tick recoveries are reported throughout the province and *B*. *burgdorferi* infection of both *I*. *scapularis* and dogs are elevated in the southern third of the province, relative to the provincial average. This broad region encompasses the areas of this study [10, 13]. No endemic or risk areas in Maritime Canada have been identified for *B*. *miyamotoi* as it is understudied due to its relative rarity in ticks in this region, relative to *B*. *burgdorferi*. The presence of *B*. *burgdorferi* reservoir competent wildlife species is well documented in Maine and Quebec [3, 14, 15] and many of these species are also present in New Brunswick. Additionally, New Brunswick has some Atlantic species of non-migratory birds and mammals not abundant in continental North America [16]. The potential for a variety of *Borrelia* species and strains to establish in the New Brunswick wildlife is further enhanced by the intersection of both trans-Atlantic and North American bird migration flyways over the province, which provides a potential source of ticks infected with non-local *Borrelia* [17–22].

The primary reservoir of *B*. *burgdorferi* in North America is the white-footed mouse (*Peromyscus leucopus*) and the closely related deer mouse (*Peromyscus maniculatus*), with overlapping ranges covering central and eastern North America [8, 23–25]. A number of other *B*. *burgdorferi* reservoir species have been identified: the eastern chipmunk (*Tamias striatus*), eastern and western grey squirrels (*Sciuris griseus*, *Sciuris carolinensis)*, and some shrew species (*Blarina brevicauda*, *Sorex cinereus*) [14, 23, 26–28]. These are animals that have been shown to competently maintain and transmit the infection to ticks, maintaining *B*. *burgdorferi* between tick generations. *B*. *miyamotoi* has been studied less than *B*. *burgdorferi* [6, 29]. Some European *B*. *miyamotoi* reservoir species have been identified [29–32] and in North America, the white-footed mouse has been identified as a *B*. *miyamotoi* reservoir [8, 25]. The deer mouse has not been studied for *B*. *miyamotoi* and is the dominant wild mouse species in Atlantic Canada. The impact of *B*. *miyamotoi* on wildlife is unknown, *B*. *miyamotoi* has a higher blood spirochete load in mammals than *B*. *burgdorferi* suggesting they inhabit different niches in the host, so may have different effects on host health [31]. While *B*. *miyamotoi* is capable of vertical transmission within ticks [7, 8], vertical transmission has not been previously shown in mammalian hosts. However vertical transmission in mammals is well documented for another relapsing fever *Borrelia*, *B*. *duttonii* [8, 33, 34].

The *Borrelia* transmission cycle requires both infected ticks and animals so to assess the risk of *Borrelia* exposure in a region, both need to be monitored [35, 36]. To investigate the incidence of *B*. *burgdorferi* and *B*. *miyamotoi* in Atlantic Canada, we used a citizen science approach to survey a wide range of animals including migratory birds, medium-sized mammals, and small mammals. This design was employed to yield a wide variety of species as opposed to a targeted approach as no previous information existed on which animals, if any, are acting as reservoirs for *Borrelia spp*. in New Brunswick. To determine the infection status of an animal, multiple tissues and organs were tested. An important consideration for an animal to be a reservoir is that there be a negligible loss of fitness from carrying the bacteria as this ensures its ability to transmit the bacteria between ticks. In human and animal models, late-stage infections among symptomatic hosts are caused by the dissemination of *B*. *burgdorferi* throughout the body [37–39]. In contrast animals with robust immune responses or antibiotic treatment show infections localised to a single organ [40, 41]. Determining the distribution of *Borrelia spp*. within the tissues of an infected animal can inform whether this animal is potentially an unaffected carrier or if they could be suffering a loss of fitness.

## Materials and methods

### Sample collection

To maximize the number and diversity of species collected two non-targeted collection approaches were used. In the summer of 2016 (May-August) medium-sized animals were collected twice-weekly through roadkill collection. All roadside specimen recovery locations were recorded using GPS, and UTM coordinates (S2 Table and S2 Fig). Small animals such as rodents, squirrels, and smaller birds, were collected by using a public participatory approach where, primarily, cat-killed specimens were provided by the public from May 2016 to December 2017. These collection approaches provided animals and tissues that were either frozen (small mammal community donations) or of varying degrees of freshness (medium sized mammals, roadside collections), which precluded culturing from the sample tissues. Mammals and birds were identified morphologically by Gay Hansen, curator of the Mount Allison university ornithology collection; specimens too damaged for morphological identification but from which tissues could still be obtained were also tested and are listed as “unknown species”. Locations for these animals were noted as the civic addresses of the donors or locations nearest to where they were found.

Roadkill dissections were performed on the roadside. Animals were surface sterilised with 70% ethanol prior to dissection and samples were held at approximately 4°C for the duration of the sampling trip until frozen at -20°C prior to DNA extraction. Small animals were stored whole at -20°C prior to dissection and then dissected under sterile conditions in the laboratory. All laboratory dissections were performed under sterile conditions in a fume hood. Animals were surface sterilised with 70% ethanol prior to dissection, and all implements were autoclaved and stored in 70% ethanol between uses and scalpel blades were discarded after single use. Dissections were performed wearing approved personal protective equipment. Tissue samples were placed in sterile microcentrifuge tubes and all specimens were stored in separate sealed plastic bags. An initial dissection was performed to remove liver and kidney samples, if present, as well as bladder, muscle, and skin samples, if present. Skin samples were taken from the ear and tail, if applicable, for mammalian specimens and from the neck and breast for avian specimens. Positive samples identified in the initial screening of kidney and liver, as well as representative negatives were dissected to obtain samples of the brain, lungs, heart, spleen, and gonads. In one instance, fetuses that were present were also collected and dissected from their amniotic sacs to obtain tissue exclusively from the fetus. Blood was not considered due to the difficulty in obtaining samples from deceased animals in variable condition. Representative negatives were species matched negative animals independent of location. In 5 cases, animals initially thought to be negative because their kidney and liver were negative but were later found to have other infected tissues. In these cases, additional species matched negatives were selected. All specimens were thoroughly inspected for ticks, but only rabbit ticks (*Haemaphysalis leporispalustris*) were found on two snow shoe hares. As ticks rapidly detach from dead hosts [42], ticks would be unlikely to be found on less infested dead hosts.

Approval for the use of tissues obtained from necropsy was given by the Animal Care Coordinator of Mount Allison University, protocol number NEC 2016–01. Approval for collection of all specimens excluding migratory birds was given through the government of New Brunswick, Fish and Wildlife branch, permit number SP16-006. Approval for collection of migratory birds was given through the Canadian Wildlife Service, permit number SS2025.

### DNA extraction

For each animal, target tissue samples were removed and stored at -20°C prior to DNA extraction. Whole DNA was extracted using the AquaGenomic^TM^ (Multi target Pharmaceuticals) tissue protocol. For all samples this procedure was performed under sterile conditions in a sterilized fume hood. Tissue samples were placed in 100μL AquaGenomic solution with proteinase K (5μg) for a 90-minute incubation at 55°C followed by a 10-minute deactivation step at 95°C. The samples were then hand vortexed for approximately 30 seconds then centrifuged at 12000Xg in a desktop microcentrifuge for 5 minutes. The supernatant was removed and mixed with an equal volume of isopropanol to precipitate the DNA, mixed and re-centrifugation at 5 minutes to collect the precipitated DNA. The pellet was rinsed with 70% ethanol and left to air dry overnight at room temperature in sterile conditions, before being resuspended in 50μL nuclease-free water (ThermoFisher) and stored at -20°C.

### Nested-PCR

Detection of *Borrelia spp*. used nested-PCR (nPCR) to enhance specificity. Outer primers (Rrs and Rrl; Table 1) designed to detect multiple *Borrelia* species were as described by DiBernardo et al. [43]. These primers target the 16-23s intergenic spacer, a conserved region of the *Borrelia* genus, and detect multiple Lyme and relapsing fever *Borrelia* species [43]. Inner primers specific for *B*. *burgdorferi* and *B*. *miyamotoi* were then designed and used (Burg23S and Miya23S; Table 1). All nPCR reactions were set up in a surface- and UV-sterilized laminar hood and performed in 0.5mL tubes in a Labnet MultiGene OptiMax Thermal Cycler with 12.5μL GoTaqGreen polymerase (Promega), 8.5μL of nuclease free water, 1μL of the forward and reverse primers (Sigma), and 2μL of sample DNA. Negative controls, pre and post-reaction, in which animal DNA was replaced by nuclease free water were performed and animal dissection, DNA extraction, PCR reactions and gel electrophoresis were all performed in different work spaces with independent air flow. The amplification program for the initial reaction was 4 min at 94°C, 35 cycles of 94°C 1 min, 50°C 1 min, 72°C 1 min, followed by 72°C for 10 min [43]. The second round of amplification was performed as described above: 5 min at 95°C, 40 cycles of 95°C 30 sec, annealing temperature (Table 1) 30 sec, 72°C 30 sec, followed by 72°C for 10 min. PCR products were size-separated by agarose gel electrophoresis (1.2% agarose in sodium borate buffer). The liver and kidney were targeted initially from each specimen for *B*. *burgdorferi* and *B*. *miyamotoi*. A positive from either tissue meant that the animal was considered positive. All positive amplicons were confirmed by Sanger sequencing through Genome Quebec, McGill University, Canada.

**Table 1:** Primer sequences and their associated amplicon sizes.

Primer name	5’-3’ Sequence	Annealing Temperature	Amplicon	source
Rrs (outer)	GTATGTTTAGTGAGGGGGGTG	50°C	588-1029bp	[43]
Rrl (outer)	GGATCATAGCTCAGGTGGTTAG			
Burg23S_Inner_F	ATGTATTCCATTGTTTTAATTACG	51°C	340bp	[44]
Burg23S_Inner_R	GACAAGTATTGTAGCGAGC			
Miya23S_Inner_F	ATAAACCTGAGGTCGGAGG	60°C	447bp	[44]
Miya23S_Inner_R	AAAGTGTGGCTGGATCACC			

### Sensitivity and specificity testing of *B*. *burgdorferi* detection

All amplicons produced during testing were sequenced to assess primer specificity. Both primer sets created in this project were sequence-validated for use in each of the species tested. While not every species had a representative positive, there were no false positives found through sequencing, indicating that the primers do not cross-react with any of the mammalian or avian host species’ DNA. Amplification sensitivity was assessed by spiking negative animal DNA samples with DNA isolated from pure *B*. *burgdorferi* B31 culture (ATCC #35210). The spiked DNA was assessed by nPCR as described above. DNA concentration was measured using a NanoDrop 1000 Spectrophotometer (ThermoFisher). The number of genomes present was calculated based on the total genome size, including plasmids, of *B*. *burgdorferi* strain B31 chromosome on Genbank (Accession: NC_001318). No sensitivity testing was performed for the *B*. *miyamotoi* detection as no commercial culture or whole genome DNA was available.

### Sequence and phylogenetic analysis

**Fig 1:**
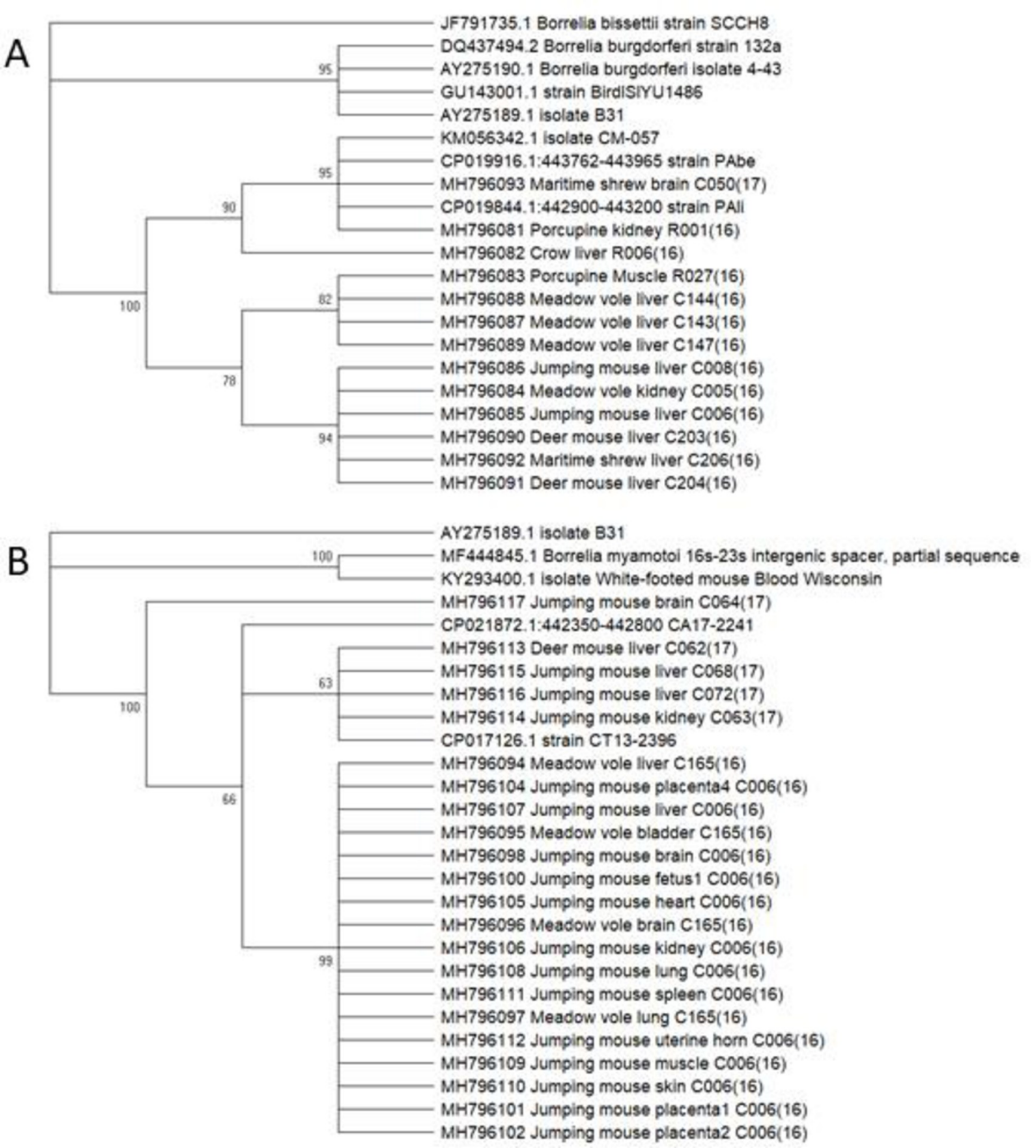
Phylogenetic alignments for *B*. *burgdorferi* and *B*. *miyamotoi* aligned sequences. *B*. *burgdorferi* (A) and *B*. *miyamotoi* (B) aligned sequences were run through Clustal Omega to produce phylogenetic trees. Additional sequences retrieved from Genbank were included in the trees. All sequences have their accession number, and where they come from the same specimen are indicated with their sample number (e.g. C006). Both trees are after 1000 bootstrap iterations and have any branches produced in less than 50% of the iterations removed. The *B*. *burgdorferi* tree (A) is rooted on *B*. *bissettii* (strain SCCH8) and the *B*. *miyamotoi* tree (B) is rooted on *B*. *burgdorferi* (strain B31).

To confirm sequences, FinchTV software (FinchTV 1.4.0, Geospiza, Inc.; Seattle, WA, USA; http://www.geospiza.com) was used to manually compare opposite direction reads and resolve any discrepancies through reference to the original chromatogram. An nBLAST analysis of the NCBI Genbank database [45] nucleotide collection (nr/nt) with no exclusions or organismal selection was performed selecting for highly similar sequences (megablast), most recently on October 15, 2018. Only sequences confirmed in both directions were used to identify sequences with the closest match to the amplified product (S1 Table). Amplified sequences from all *Borrelia*-positive samples have been deposited in the Genbank database; accession numbers, sequences and nBLAST results are indicated in S1 Table. Maximum-likelihood phylogenetic trees were constructed following the protocol established by Hall [46]. Both trees (Fig 1A and 1B) included outgroups and reference sequences for comparison retrieved from Genbank. To build the trees, first model selection was run using Mega X [47], with partial deletion and a 95% site coverage cutoff. For the *B*. *miyamotoi* tree, two sequences (MH796099 & MH796103) from different tissues of the same organism were omitted, as the overall analysis is constrained by the shortest sequence included. Both trees (Fig 1A and 1B) were based on the Tamura-Nei model [48] and bootstrapped for 1000 replicates [49]. The *B*. *burgdorferi* tree was rooted on *B*. *bissettii* strain SCCH8 (Genbank accession #JF791735.1), while the *B*. *miyamotoi* tree was rooted on *B*. *burgdorferi* strain B31 (Genbank Accession #NC_001318.1). Both trees are shown with any branch appearing in less than 50% of the bootstrap replicates collapsed.

### Data analysis

Spatial trend analysis was done using ArcMap (ESRI 2015). Samples were plotted with their location of recovery and infection status for both *B*. *burgdorferi* and *B*. *miyamotoi*. A Getis-Ord spatial clustering analysis was done for both *Borrelia* species [50]. Determination of any species predictive of *Borrelia* infection was done by logistic regression using Rstudio (2016) and the package ROCR [51] to determine if there were species with a higher likelihood of infection than others.

## Results

### *B*. *burgdorferi* and *B*. *miyamotoi* in wildlife species

The goal of this study was to identify *Borrelia* infected species in New Brunswick, assess the distribution of *Borrelia* in their tissues as an indirect way to gauge their ability to act as pathogen reservoirs and, lastly, to determine if they were present outside of known endemic areas. As such, collection methods with as little species bias as possible were required. Use of vehicle- and cat-killed specimens was successful in obtaining a plentiful and diverse collection of species (Table 2).

**Table 2:** Number and species of animals with sequence-confirmed *B*. *burgdorferi* and *B*. *miyamotoi*.

Species	Scientific name	Number sampled	*B*. *burgdorferi*^*a*^	*B*. *miyamotoi*^*a*^
Meadow vole	*Microtus pennsylvanicus*	146	4 (2.7%)	1 (0.7%)
Deer mouse	*Peromyscus maniculatus*	34	2 (5.9%)	1 (2.9%)
Jumping mouse^*b*^	*Napaeozapus insignis*	21	2 (9.5%)	3 (14.3%)
Eastern grey squirrel	*Sciuris carolinensis*	4	0 (0%)	1 (25%)
Shrew	*Sorex maritimensis*	28	2 (7.1%)	0 (0%)
Porcupine	*Erethizon dorsatum*	21	2 (9.5%)	0 (0%)
American crow	*Corvus brachyrhynchos*	11	1 (9.1%)	0 (0%)
Chipmunk	*Tamias striatus*	2	0 (0%)	0 (0%)
Brown rat	*Rattus norvegicus*	9	0 (0%)	0 (0%)
Short tailed weasel	*Mustela erminea*	1	0 (0%)	0 (0%)
Raccoon	*Procyon lotor*	9	0 (0%)	0 (0%)
Groundhog	*Marmmota monax*	4	0 (0%)	0 (0%)
Snow shoe hare	*Lepus americanus*	5	0 (0%)	0 (0%)
Red fox	*Vulpes vulpes*	1	0 (0%)	0 (0%)
Red squirrel	*Tamiasciurus hudsonicus*	3	0 (0%)	0 (0%)
Muskrat	*Ondatra zibethicus*	2	0 (0%)	0 (0%)
Common garter snake	*Thamnophis sirtalis*	1	0 (0%)	0 (0%)
Mallard	*Anas platyrhynchos*	1	0 (0%)	0 (0%)
American black duck	*Anas rubripes*	1	0 (0%)	0 (0%)
American robin	*Turdus migratorius*	1	0 (0%)	0 (0%)
Ruffed grouse	*Bonasa umbellus*	2	0 (0%)	0 (0%)
Rock pigeon	*Columba livia*	2	0 (0%)	0 (0%)
Belted kingfisher	*Megaceryle alcyon*	1	0 (0%)	0 (0%)
Common redpoll	*Acanthis flammea*	3	0 (0%)	0 (0%)
Pine siskin	*Spinus pinus*	2	0 (0%)	0 (0%)
American goldfinch	*Spinus tristis*	1	0 (0%)	0 (0%)
House finch	*Haemorhous mexicanus*	1	0 (0%)	0 (0%)
Downy woodpecker	*Picoides pubescens*	1	0 (0%)	0 (0%)
Ruby-throated Hummingbird	*Archilocus colubris*	1	0 (0%)	0 (0%)
Unknown	Unknown	20	0 (0%)	0 (0%)
	**Totals**	339	13 (3.8%)	6 (1.8%)

^a^Percent of specimens infected shown in parentheses

^b^One co-infected jumping mouse is represented as a *B*. *burgdorferi* and a *B*. *miyamotoi* positive

The primers developed to detect *B*. *burgdorferi* and *B*. *miyamotoi* for this study were specific; amplicons of the expected size yielded *Borrelia* sequences and there were no false positives found through sequencing, indicating that the primers do not cross-react with any of the mammalian or avian host species’ DNA (S1 Table). Host-derived amplicons of smaller sizes were observed, but readily distinguishable from *Borrelia*-derived sequences by size (S2 Fig). While *Borrelia* was not found in every host species, sensitivity tests were performed on the *Borrelia burgdorferi* specific inner primers using negative host animal DNA spiked with *B*. *burgdorferi* B31 DNA. Reliable detection occurred with as few as 100 genomes *B*. *burgdorferi* added, or 50 genomes/μL of sample DNA. The ratio of *B*. *burgdorferi* genomes to wood thrush (*Hylocichla mustelina*, avian test sample) genomes at reliable detection was 1:3,090. The ratio of *B*. *burgdorferi* genomes to meadow vole (*Microtus pennsylvanicus*, mammalian test sample) at reliable detection was 1:13,200. This indicates that this protocol is able to detect few *Borrelia* genomes despite the overwhelming presence of host DNA. Further, coinfection of *B*. *miyamotoi* and *B*. *burgdorferi* was able to be simultaneously detected, although the presence of multiple *B*. *burgdorferi* strains could confound sequencing.

Over the two-year study period, a total of 339 animals were obtained and tested. Every animal had a minimum of two tissues tested by nPCR for both *B*. *burgdorferi* and *B*. *miyamotoi*. *B*. *burgdorferi* and *B*. *miyamotoi* were found in a wide range of mammalian host species: meadow vole, deer mouse, eastern grey squirrel, jumping mouse, shrew, porcupine, and American crow. Of these 339 animals, 13 had sequence-confirmed *B*. *burgdorferi* infections, 6 had sequence-confirmed *B*. *miyamotoi* infection, and one animal (jumping mouse) was infected with both *Borrelia* species (Table 2).

Sequence analysis of both *B*. *burgdorferi* and *B*. *miyamotoi* amplicons showed that all differed slightly and clustered with each other more closely than with *B*. *burgdorferi* strain B31 (Fig 1), as would be expected for wild-derived samples. There was no obvious clustering by host species or host tissue, which is not unexpected for a pathogen transmitted by a non-species-specific vector. The 16-23S intergenic spacer is a non-coding region which allows for it to have highly variable sections. As such conclusions cannot be made as to the presence of different strains from these sequences.

### *Borrelia* surveillance

The most frequently *B*. *burgdorferi*-infected species were porcupine (2/21), crow (1/11), and jumping mouse (2/21), all with approximately 9.5% of individual specimens with sequence-confirmed *B*. *burgdorferi*. For *B*. *miyamotoi*, the jumping mouse was the species with the highest *Borrelia* detection at 3/21 (14.2%). To determine if specific species were more likely to have an infection, we used logistic regression, separately for *B*. *burgdorferi* and *B*. *miyamotoi*. The variable numbers obtained for each species, as well as the low representation of some species reduced the power of statistical analysis. For *B*. *burgdorferi*, a logistic regression model was created with a McFadden r^2^ of 0.098, an accuracy based on ROC curve of 0.734, and no significant species found. The model for *B*. *miyamotoi* was similar with a McFadden r^2^ of 0.292, an accuracy based on ROC curve of 0.899, and no significant species found. A targeted approach directed at specific species would allow more robust statistical analysis to identify a single predictive species, however, this was not the goal of this study.

Having no single significant predictive species allows all the samples to be used in the spatial analysis to determine if infected animals were clustered, potentially representing an isolated endemic area, or non-clustered, representing many isolated or a large region of bacterial penetration into wildlife. To test spatial clustering of *B*. *burgdorferi* and *B*. *miyamotoi* positive samples, a Getis-ord General G was calculated. This is a measure of spatial autocorrelation to determine whether high or low values are clustered [50]. In this context, it is measuring whether positive samples are clustered or randomly distributed. This was calculated using a fixed-Euclidean distance as a conservative measure. Spatial analysis was done separately for *B*. *burgdorferi* and *B*. *miyamotoi*. *B*. *burgdorferi* had a p-value of 0.426. This distribution of positives does not differ from random so there are no clusters. For *B*. *miyamotoi*, a p-value of 0.088 was found. While not significant at a threshold of 0.05, when taken with a positive z-score (z-score = 0.796109) suggests a trend towards clustering positives. In both cases, resolution of this analysis is limited by the non-random sample collection; medium-sized animals were recovered from roadways and smaller samples originated primarily from the southeastern region of New Brunswick near the university. Nevertheless, for both *B*. *burgdorferi* and *B*. *miyamotoi*, the lack of significant clustering means that infected animals were not restricted to single geographic areas and were, instead, widely dispersed.

### Tissue distribution of *Borrelia*

**Fig 2:**
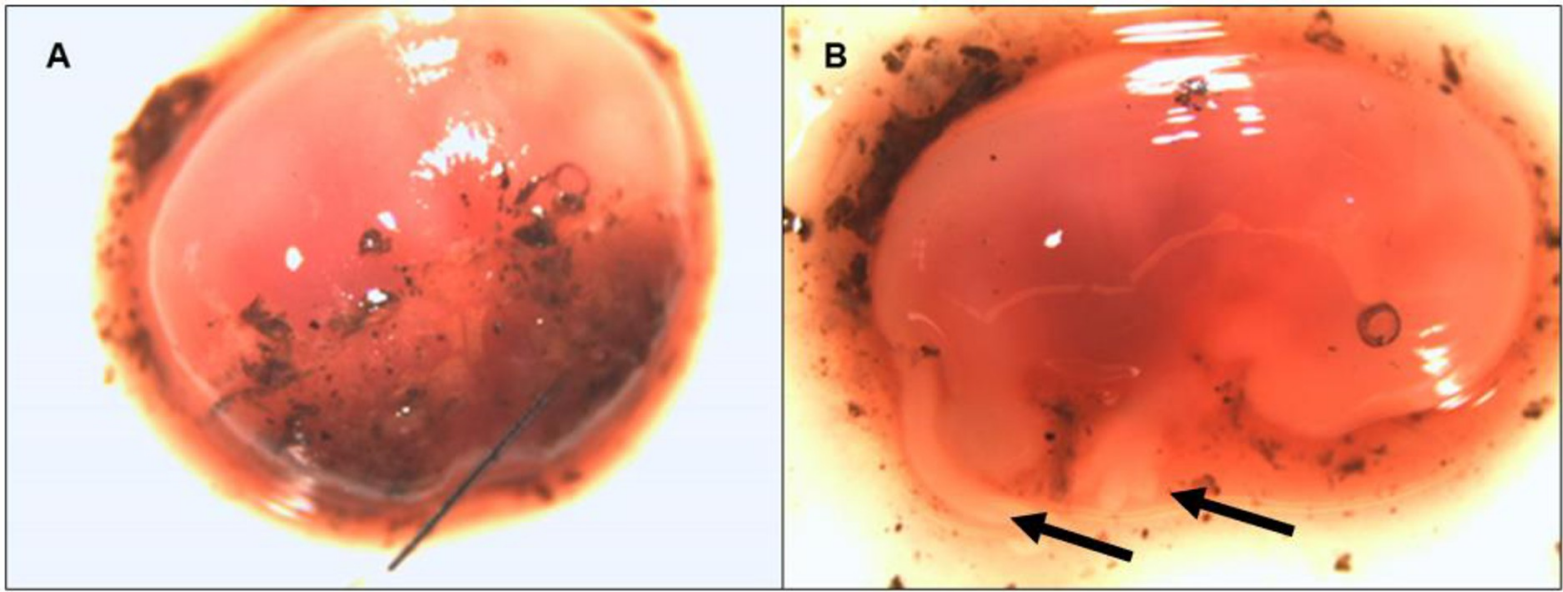
Two late stage jumping mouse fetuses removed for extraction and testing. (A) Still in the amniotic sac and with attached placenta (left; thin line on right is a dissecting pin). (B) Removed from the amniotic sac and separated from placenta. Both fetuses have separation between their digits, and resolved tails, indicated with arrows.

Fourteen animals, representing 5 species (Table 3), identified as having *B*. *burgdorferi* and/or *B*. *miyamotoi* based on testing of liver and kidney, along with negative specimens of the same species, then had additional target tissues tested to assess in what tissues and organs the spirochetes could be found. For both *B*. *burgdorferi* and *B*. *miyamotoi*, the liver was the most common tissue found infected, and was the most common single organ to be found infected in an animal. For both *B*. *burgdorferi* and *B*. *miyamotoi*, one animal was found where the infection was exclusively detected in the brain; a shrew infected with *B*. *burgdorferi* and a jumping mouse infected with *B*. *miyamotoi*. All identified *B*. *burgdorferi* infections were in a single tissue/organ whereas there were two instances of diffuse infection for *B*. *miyamotoi*. One of these animals was a female, pregnant, jumping mouse (*Napaeozapus insignis*) co-infected with *B*. *burgdorferi* and *B*. *miyamotoi*. For this animal, in addition to the tissues listed in Table 3, two late development fetuses (Fig 2) were removed and tested separately for the presence of both bacteria. *B*. *miyamotoi* was found in the placentae and in both fetuses.

**Table 3:** Tissues tested for each animal. Each animal was tested for *B*. *burgdorferi* and *B*. *miyamotoi* with positives denoted as “B” or “M”, respectively. All specimens were from New Brunswick or Cumberland County, Nova Scotia, which directly borders New Brunswick. The year of collection is given in the second column.

Species	Year	Liver	Bladder	Kidney	Muscle	Skin	Brain	Heart	Spleen	Lung	Uterine horn	Testes
Deer mouse^2^	2016	**B**	n/a	-	-	-	-	-	-	-	-	n/a^1^
Deer mouse^2^	2016	**B**	-	-	-	-	-	-	-	-	-	n/a
Deer mouse	2017	**M**	-	-	-	-	-	-	-	-	n/a	n/a
Deer mouse	2017	-	-	-	-	-	-	-	-	-	n/a	n/a
Deer mouse	2016	-	-	-	-	-	-	-	-	-	n/a	-
Deer mouse	2016	-	-	-	-	-	-	-	-	-	n/a	n/a
Eastern grey squirrel	2017	**M**	-	-	-	-	-	-	-	-	n/a	n/a
Eastern grey squirrel	2017	-	-	-	-	-	-	-	-	-	n/a	n/a
Eastern grey squirrel	2017	-	-	-	-	-	-	-	-	-	n/a	n/a
Jumping mouse	2016	**MB**	-	**M**	**M**	**M**	**M**	**M**	**M**	**M**	**M**	n/a
Jumping mouse	2016	**B**	-	-	-	-	-	-	-	-	-	n/a
Jumping mouse	2017	-	-	**M**	-	-	-	-	-	-	n/a	n/a
Jumping mouse	2017	**M**	-	-	-	-	-	-	-	-	n/a	n/a
Jumping mouse	2017	-	-	-	-	-	**M**	-	-	-	n/a	n/a
Jumping mouse	2016	-	-	-	-	-	-	-	-	-	n/a	n/a
Jumping mouse	2016	-	-	-	-	-	-	-	-	-	n/a	n/a
Jumping mouse	2017	-	-	-	-	-	-	-	-	-	n/a	n/a
Jumping mouse	2017	-	-	-	-	-	-	-	-	-	n/a	n/a
Meadow vole	2016	-	-	**B**	-	-	-	-	-	-	-	n/a
Meadow vole	2016	**B**	-	-	-	-	-	-	-	-	-	n/a
Meadow vole	2016	**B**	-	-	-	-	-	-	-	-	-	n/a
Meadow vole	2016	**B**	-	-	-	-	-	-	-	-	-	n/a
Meadow vole	2016	**M**	**M**	-	-	-	**M**	-	-	**M**	-	n/a
Meadow vole	2017	-	-	-	-	-	-	-	-	-	n/a	-
Meadow vole	2016	-	-	-	-	-	-	-	-	-	n/a	n/a
Meadow vole	2016	-	-	-	-	-	-	-	-	-	n/a	-
Meadow vole	2016	-	-	-	-	-	-	-	-	-	n/a	-
Shrew^2^	2016	**B**	-	-	-	-	-	-	-	-	-	n/a
Shrew	2017	-	-	-	-	-	**B**	-	-	-	n/a	n/a
Shrew	2017	-	-	-	-	-	-	-	-	-	n/a	n/a
Shrew	2017	-	-	-	-	-	-	-	-	-	n/a	n/a
Shrew	2016	-	-	-	-	-	-	-	-	-	n/a	n/a

n/a = not applicable. Indicates that uterus/testes only present in female or male animals, respectively.

Denotes animals that originated in the nearby Cumberland County, Nova Scotia.

## Discussion

This study reports *B*. *burgdorferi* and *B*. *miyamotoi* in multiple members of five non-migratory small mammal species within New Brunswick, including the jumping mouse, a species not previously identified as an important reservoir species. We found that *B*. *miyamotoi*, unlike *B*. *burgdorferi*, was found disseminated into multiple organs and we documented the first evidence of trans-placental transmission of *B*. *miyamotoi* in mammals. In order to maximize number and diversity of animal species collected, we used a public participatory or citizen/community science approach. An important advantage of this approach is that it allows collection of a large number of specimens with limited cost. By collecting road and cat-killed specimens, we also avoided the intentional sacrifice of animals. However, such non-targeted collection methods have disadvantages, in this case resulting in geographically uneven collections and uneven distributions of samples among species. These approaches also could have been biased towards recovery of slower, ill, or reckless individuals.

### Borrelia burgdorferi

*B*. *burgdorferi* is expected to be found in a variety of wildlife species. However, the Atlantic region is home to some species not abundant in the rest of Canada, and equally, lacks species common in central and southern parts of North America [16] and there has been no investigation of which species would be suitable for ongoing surveillance in the Canadian Maritimes. The most common North American reservoir species is the white-footed mouse (*Peromyscus leucopus*), but this species is rare in New Brunswick. A close relative, the deer mouse (*Peromyscus maniculatus*) is abundant, however, and both the deer mouse and white-footed mouse have similar reservoir capabilities [23, 31, 52, 53]. Site studies in American locations with high incidences of Lyme disease but only small populations of white-footed mice have shown the deer mouse [53], and the meadow vole (*Microtus pennsylvanicus*) [54] to be the principal reservoirs of *B*. *burgdorferi*. Other species found to be reservoirs are the eastern chipmunks, eastern grey squirrels, and short-tailed shrews [14, 36]. These species are abundant in New Brunswick. One species not previously identified as either a reservoir species or one having a high infection prevalence is the jumping mouse, *Napaeozapus insignis*. While Anderson et al. [26] did note that infected jumping mice have been found, in our study this species had the highest prevalence of infection for both *B*. *burgdorferi* and *B*. *miyamotoi* singly, as well as a double infection.

### Borrelia miyamotoi

*Borrelia miyamotoi* was first discovered in Japan in 1995, and in North America in 2001 and since detected in humans, ticks, and wildlife worldwide [8, 31, 55]. Currently *B*. *miyamotoi* reservoir species are believed to be primarily the same as *B*. *burgdorferi* [56]. Our findings support this distribution as the only animal in which we identified *B*. *miyamotoi* that had not been previously described as a reservoir species was, again, the jumping mouse *Napaeozapus insignis*. *B*. *miyamotoi* has been documented in ticks found across Canada, with an approximately 1% infection prevalence in New Brunswick as of 2012 [43]. Our identification of *B*. *miyamotoi* in ~2% of a variety of wildlife species from different regions of New Brunswick in 2016/2017 indicates that *B*. *miyamotoi*, along with *B*. *burgdorferi*, is established in wildlife in the province. While it is not currently as prevalent as *B*. *burgdorferi*, proliferation in the wildlife, particularly if vertical transmission is possible, will lead to an increase in the likelihood of human and companion animal infections.

While most of the species found in this study have been shown to be *B*. *burgdorferi* reservoirs [8, 32, 36], as our specimens were collected primarily as road or cat kill, this study could not directly test the ability of any given species to transmit infection to a feeding tick and so cannot show formally that these species are acting as reservoirs. This question can, however, be addressed indirectly by looking at tissue distribution of the *Borrelia* infections.

### Tissue distribution of *Borrelia*

In our study, the liver and kidney were targeted for initial screening. The liver was chosen as the primary organ for direct bacterial detection due to it being a common site of disseminated *B*. *burgdorferi* infection in experimental models, as well as a common refuge tissue for immune evasion in long-term infections [57]. It has also been used as a source organ for direct culturing of live *B*. *burgdorferi* [36, 56]. The kidney is similarly an organ often used for the culturing of live *B*. *burgdorferi* spirochetes [26, 36, 56, 58]. In human and animal models, the dissemination of *B*. *burgdorferi* is thought to be dependent on time since infection. At the onset of infection, the bacteria localise to the skin and tissue surrounding the site of the tick bite. The bacteria then disperse systemically through the circulatory and lymphatic systems before they begin to extensively replicate and colonise organs [37–39]. This is followed by the late disseminated stage, where bacteria are detectable in numerous systems and reflect failure of the host immune system to control the *B*. *burgdorferi* infection. In either host species with robust immune responses or late-stage infection hosts treated with antibiotics, *B*. *burgdorferi* persistence in refuge tissues is commonly observed [40, 41]. This persistence is thought to entail *B*. *burgdorferi* evading the immune response and antibiotic treatment by adopting less metabolically active states in one or few organs. In mice models of infection, the liver was a common refuge tissue for *B*. *burgdorferi* where the bacteria evaded Kupffer cells, and thus the host immune response [41]. However, this same study also reported the highest levels of *B*. *burgdorferi* being found in the liver within 24 hours of injection into the bloodstream [41]. Since blood is filtered through the liver it is a likely organ for initial infection when *B*. *burgdorferi* leaves the circulatory system.

Of the ten *B*. *burgdorferi* positive animals found in this study, eight had an infection only detectable in the liver. This means that these animals were either killed within 24 hrs of the *B*. *burgdorferi* reaching the dissemination phase from a natural infection via tick bite, or, more likely, they are in a persistent late infection stage in which *B*. *burgdorferi* are confined to the refuge tissue to evade the host immune response. The other two infected animals also showed only one infected tissue, a meadow vole (*Microtus pennsylvanicus*) with the infection present in a kidney, and a shrew (*Sorex maritimensis*) with infection confined to the brain (Table 3). The single-organ distribution of infected animals in this study, as well as the narrow window of localized distribution early in infection suggests that most of the animals recovered were likely at the long-term persistent infection stage; the host immune response was keeping the bacteria from complete dissemination through the body, but not able, or yet able, to eliminate it completely.

In humans, lab mice (*Mus musculus*), rhesus macaques, dogs, and horses, *B*. *burgdorferi* and *B*. *miyamotoi* infection causes a range of symptoms, from erythema migrans to arthritis and carditis [4, 40, 57, 59]. The functional effects of infection can be obtained or inferred from patient reports of symptoms, supplemented with serological findings and sometimes culturing or direct molecular detection in blood or cerebrospinal fluid [4, 60]. Assessment by biopsy or autopsy is rarely performed [61]. In animal disease models, effects of infection are monitored based on health cues such as weight and activity level, and with *B*. *burgdorferi* the swelling of joints is a common measurement in mice [24, 37, 40]. Terminal tissue testing can be used to correlate the presence of infection in the tissues with physiological and histological signs of disease. Correlating this information with the location of infection, inferences can be made as to the effects of infection on the biologically relevant host animals in the wild, although such extrapolations are not straightforward. In human and non-human models such as *Mus musculus*, even after antibiotic intervention to remove the majority of the *B*. *burgdorferi* infection, symptoms can persist, which has been attributed to a number of causes, including persistent infection, physical damage arising from infection, or autoimmune response to bacterial proteins [59, 62, 63].

In contrast, the white-footed and deer mouse can both maintain a persistent infection and mount an immune response without obvious effects [24, 25, 64]. For other wildlife species, it is not known if infection impairs health, as in *Mus musculus*, or has no observable effect as reported for *Peromyscus spp*.; capturing and experimentally infecting a large number of species of wild animals is impractical. Additionally, with wild animals, it is impossible to be certain that signs of disease are caused by the *Borrelia* infection or arise from some other infection or pre-existing condition. Thus, cautious extrapolations from the location of infection is the most practical way of inferring physiological and functional effect of infection on wildlife health. The distribution of *Borrelia spp*. within an infected animal can be used to infer whether the infection is active, suggesting an ineffective immune response, or contained in a single organ. If an animal is negatively affected by the bacterial infection, it will reduce their effectiveness as a reservoir host by increasing their mortality. If they are affected, this could lead to the number of presumed late-stage infections being over represented in this study as they are more likely to be killed. If they are unaffected, they could function as ideal reservoirs, similar to the white-footed mouse, and be more capable of transmitting the infection to ticks.

Unlike *B*. *burgdorferi*, there is little pre-existing information on *B*. *miyamotoi* infection progression in wildlife or animal models. *B*. *miyamotoi* is known to cause recurrent spirochetemia, and serious febrile illness and arthritic symptoms in humans and lab mice, but its effects on wildlife is not well described [56]. *B*. *miyamotoi* is thought to share the same reservoirs as *B*. *burgdorferi*, however, the bacteria are present in greater concentrations in the mammalian blood than *B*. *burgdorferi*, similar to many relapsing fever group *Borrelia* [6, 31, 56]. In this study, seven animals were found infected with *B*. *miyamotoi*, of these five had the infection present in a single tissue (Table 3). These animals include three jumping mice, having infection solely in the brain, liver, and kidneys. Additionally, a deer mouse and an eastern grey squirrel also had the infection only present in the liver. If *B*. *miyamotoi* exhibits the similar immune evasion strategies to *B*. *burgdorferi* [65], this would suggest that these animals also represent persistent late disseminated infections associated with a host immune response preventing fully disseminated infection, but not having eliminated infection. Confirmation would require controlled infections to be studied in captive populations of each host species. In addition to animals with single-organ infection, a jumping mouse and meadow vole showed systemic infections of *B*. *miyamotoi*. Whether this occurred because of the stage of infection or because the specific host was unable to control the infection is a question of ecological significance that requires further study. In the single *B*. *miyamotoi*-infected meadow vole identified, the bacteria was detected in the liver, bladder, brain, and lungs. This would suggest that *B*. *miyamotoi* was able to cross the blood brain barrier as well as establish in less vascularised tissue such as the bladder. In this study, *B*. *miyamotoi* was found in the brains of three infected animals, showing a capability to cross the blood-brain barrier, as seen in human cases [1]. The infected jumping mouse with a systemic *B*. *miyamotoi* infection was also co-infected with *B*. *burgdorferi*. In this specimen *B*. *miyamotoi* was found throughout the animal, but *B*. *burgdorferi* was only present in the liver. In addition to the maternal tissues, two late-development fetuses, and their placentae, were extracted and tested separately for the presence of both bacteria. *B*. *miyamotoi* was found in the placentae and in both fetuses of this jumping mouse. Finding *B*. *miyamotoi* in two fetuses shows that it can also cross the transplacental barrier, passing from the infected mother to its offspring. It is unknown whether this infection would have led to a viable birth, as the effects of the systemic infection are unknown. Vertical transmission has been well documented with the relapsing fever *Borrelia duttonii* where it can lead to spontaneous abortion or infant death after birth in humans [33, 34]. The body-wide infection of the mother suggests that *B*. *miyamotoi* is successfully propagating and has not been sequestered by the host immune response. Whether this systemic infection reduces host fitness is necessary work in understanding *B*. *miyamotoi* and how it will spread through and affect wildlife. If *B*. *miyamotoi* vertical transmission is common, there will be enhanced spread of this pathogen in wildlife populations. Additionally, if transplacental transmission is a property of *B*. *miyamotoi* infections, there are veterinary and human medical implications.

### Surveillance tools for *B*. *burgdorferi* and *B*. *miyamotoi* detection in wildlife

New Brunswick reports a low incidence of human Lyme disease compared to the bordering provinces and states. The low number of human cases of Lyme disease might suggest that *B*. *burgdorferi* is not present in the local wildlife, with adventitious ticks accounting for the observed tick and human infections. However, in our study, we detected multiple infected wildlife species. Further, all infected animals detected in this study were from regions far removed from the identified endemic sites, although within zones designated as *I*. *scapularis* and human Lyme disease risk areas [66]. Both *B*. *burgdorferi* and *B*. *miyamotoi* are known to be present in ticks across New Brunswick [43, 66]. As well canine infections are prevalent through the province [10, 67]. Our findings of multiple members of reservoir-competent species infected with *B*. *burgdorferi* and *B*. *miyamotoi* demonstrate that *Borrelia* is established in New Brunswick wildlife populations and emphasizes the value of broader risk areas rather than discrete endemic areas in public health messaging.

As the risk of Lyme disease for human and companion animals arises from the presence of both ticks and infected wildlife reservoirs to infect the ticks, surveillance efforts need to monitor both components of Lyme disease risk. The primers described here allow for a rapid effective surveillance from necropsy tissue of both mammalian and avian wildlife species for *B*. *burgdorferi* or *B*. *miyamotoi*. These primers did not produce spurious amplification of the targeted sizes in any of the animals tested, and in the one instance of coinfection, they successfully amplified DNA from both bacterial species, with no cross-reaction. Further, this approach takes advantage of a public participatory citizen science model and so allows inexpensive and high-density sampling of a region. It directly detects bacterial DNA so, as opposed to serology, distinguishes between current and previous infections. However, this method is invasive and thus necessarily limited to necropsy tissue, prohibiting further study of individual animals. While effective for broad surveillance and initial investigations in an area, species-specific surveillance, using the candidate species identified in other works and this one, would be good targets for future surveillance to hone risk predictions.

## Conclusions

This study identifies the meadow vole, deer mouse, eastern grey squirrel, jumping mouse, shrew, porcupine, and American crow as candidate species to be studied for their role in carrying *Borrelia spp*. in Atlantic Canada. As well, a new species of interest, the jumping mouse, has been identified. As a preliminary step in determining if these species are able to function as *Borrelia* reservoirs and how these *Borrelia* might affect host health, we tested multiple organs and tissues from infected individuals. All ten of the *B*. *burgdorferi*-infected animals tested had the bacteria present in a single tissue, either the liver, kidney, or brain. Five of the seven *B*. *miyamotoi*-infected animals also only had a single positive tissue, either the liver, kidney, or brain. This single-site of infection suggests late stage of infection where the bacteria persists in refuge tissues and that the bacteria are not rapidly cleared could indicate that the species could act as reservoir species. In addition, two of the seven *B*. *miyamotoi*-infected animals showed widely disseminated infections, including transplacental transmission of *B*. *miyamotoi* in one jumping mouse. This discovery raises the question of whether *B*. *miyamotoi* is capable of vertical transmission, a question with both ecological and veterinary and human health implications. Our findings clearly demonstrate that the *B*. *burgdorferi* and *B*. *miyamotoi* are broadly present in non-migratory wildlife in this region of Atlantic Canada suggesting that these *Borrelia* species and pose a risk to humans and companion animal health. The methods developed here are a resource for the detection of *B*. *burgdorferi* and *B*. *miyamotoi* in mammalian tissues.

## Supporting information

S1 Fig Sample locations and identified positives from New Brunswick for 2016 and 2017. The Tantramar region of southeastern New Brunswick (main) and Southern New Brunswick (inset) with infections for both *B*. *burgdorferi* and *B*. *miyamotoi* indicated by blue and red symbols, respectively. Black symbols indicate negative samples.
(DOCX)

S2 Fig Representative gel products showing the positive amplicons. Amplification of *B*. *burgdorferi* (top) and *B*. *miyamotoi* (bottom) sequences, indicated by the red arrows in wildlife hosts. Also visible are lower weight amplicons in other samples. None of these amplicons yielded usable sequences. Every amplicon at the correct size was sequence confirmed as *B*. *burgdorferi* or *B*. *miyamotoi*.
(DOCX)

S1 Table Top 10 nucleotide-BLAST results for each identified positive sequence. All sequences are represented by their accession number. They were assessed against the NCBI Genbank nucleotide collection, specifying for highly similar sequences (megablast). Each positive tissue was sequenced resulting in some animals having multiple sequence entries.
(DOCX)

S2 Table Location data for all samples. Roadkill, denoted with an “R” sample ID, have their UTM coordinates (zone 20N) reported. The donated samples, denoted with a “C” sample ID, have their community or county of origin listed to protect the privacy of donors. The infection status of each animal is indicated by a 1, meaning detection of that *Borrelia* species, or 0 indicating it was negative.
(DOCX)
