# Supplementary material for: Borrelia burgdorferi and Borrelia miyamotoi in Atlantic Canadian wildlife
Source: PLoS One. 2022 Jan 21;17(1):e0262229. doi: 10.1371/journal.pone.0262229 (PMC8782396; doi:10.1371/journal.pone.0262229)
Supplement: S1 Fig — The Tantramar region of southeastern New Brunswick (main) and Southern New Brunswick (inset) with infections for both B. burgdorferi and B. miyamotoi indicated by blue and red symbols, respectively. Black symbols indicate negative samples. (DOCX) [file pone.0262229.s001.docx]

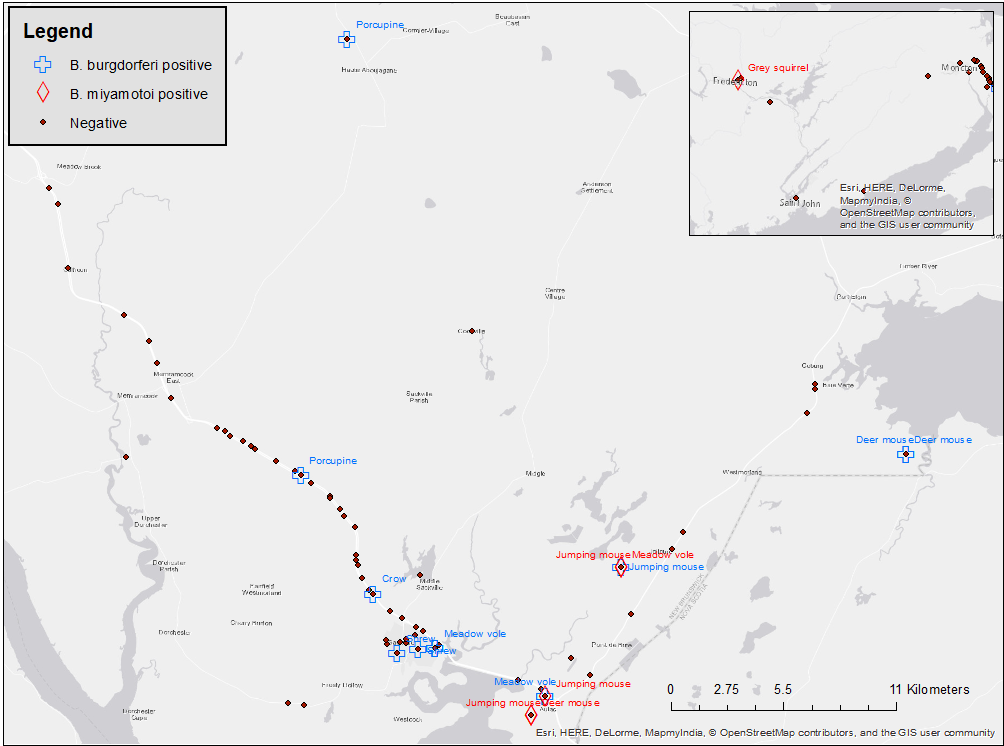


**S1 Fig.** **Sample locations and identified positives from New Brunswick for 2016 and 2017.** The Tantramar region of southeastern New Brunswick (main) and Southern New Brunswick (inset) with infections for both *B. burgdorferi* and *B. miyamotoi* indicated by blue and red symbols, respectively. Black symbols indicate negative samples.
