# Supplementary material for: Borrelia burgdorferi and Borrelia miyamotoi in Atlantic Canadian wildlife
Source: PLoS One. 2022 Jan 21;17(1):e0262229. doi: 10.1371/journal.pone.0262229 (PMC8782396; doi:10.1371/journal.pone.0262229)
Supplement: S2 Fig — Amplification of B. burgdorferi (top) and B. miyamotoi (bottom) sequences, indicated by the red arrows in wildlife hosts. Also visible are lower weight amplicons in other samples. None of these amplicons yielded usable sequences. Every amplicon at the correct size was sequence confirmed as B. burgdorferi or B. miyamotoi. (DOCX) [file pone.0262229.s002.docx]

**
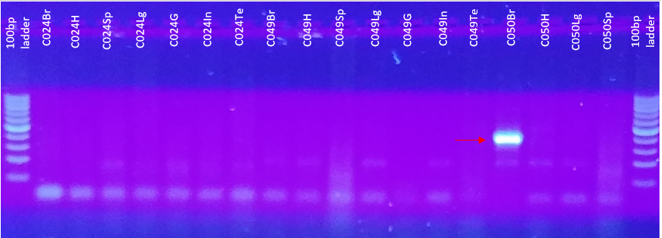

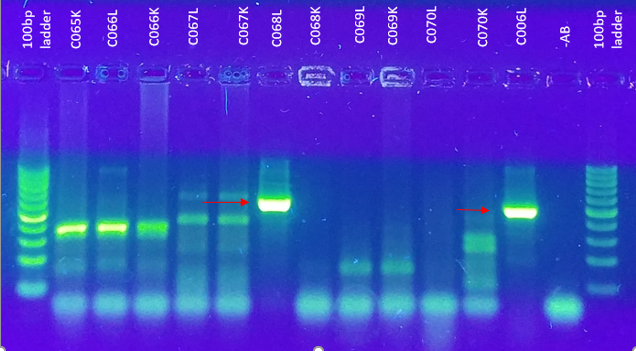
**

**S2 Fig. Representative gel products showing the positive amplicons**. Amplification of *B. burgdorferi* (top) and *B. miyamotoi* (bottom) sequences, indicated by the red arrows in wildlife hosts. Also visible are lower weight amplicons in other samples. None of these amplicons yielded usable sequences. Every amplicon at the correct size was sequence confirmed as *B. burgdorferi* or *B. miyamotoi*.
