# Supplementary material for: Borrelia burgdorferi and Borrelia miyamotoi in Atlantic Canadian wildlife
Source: PLoS One. 2022 Jan 21;17(1):e0262229. doi: 10.1371/journal.pone.0262229 (PMC8782396; doi:10.1371/journal.pone.0262229)
Supplement: S1 Table — All sequences are represented by their accession number. They were assessed against the NCBI Genbank nucleotide collection, specifying for highly similar sequences (megablast). Each positive tissue was sequenced resulting in some animals having multiple sequence entries. (DOCX) [file pone.0262229.s003.docx]

**S1 Table. Top 10 nucleotide-BLAST results for each identified positive sequence.** All sequences are represented by their accession number. They were assessed against the NCBI Genbank nucleotide collection, specifying for highly similar sequences (megablast). Each positive tissue was sequenced resulting in some animals having multiple sequence entries.

| Sample Identifier | Accession Number | Top ten Genbank matches |
| --- | --- | --- |
| R001_2016 | MH796081 | 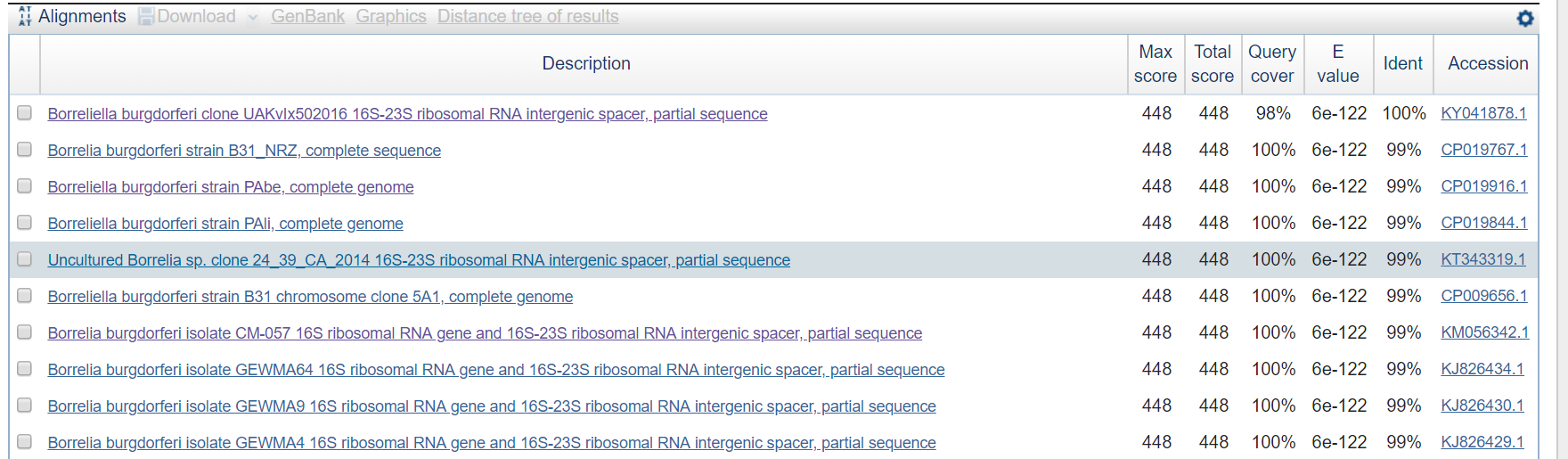 |
| R006_2016 | MH796082 | 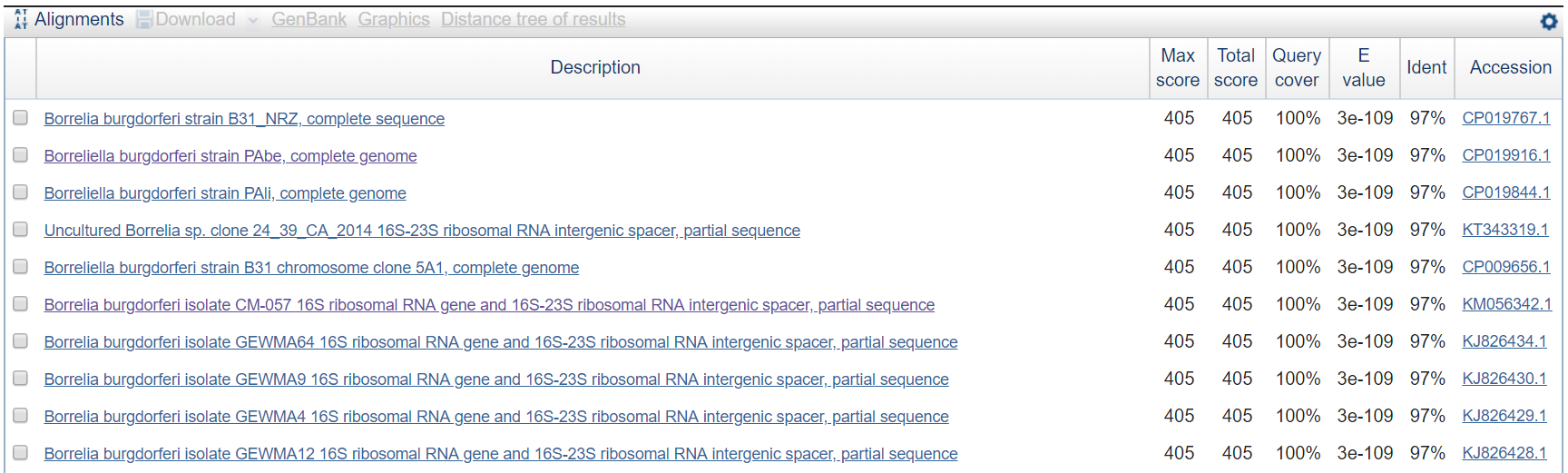 |
| R027_2016 | MH796083 | 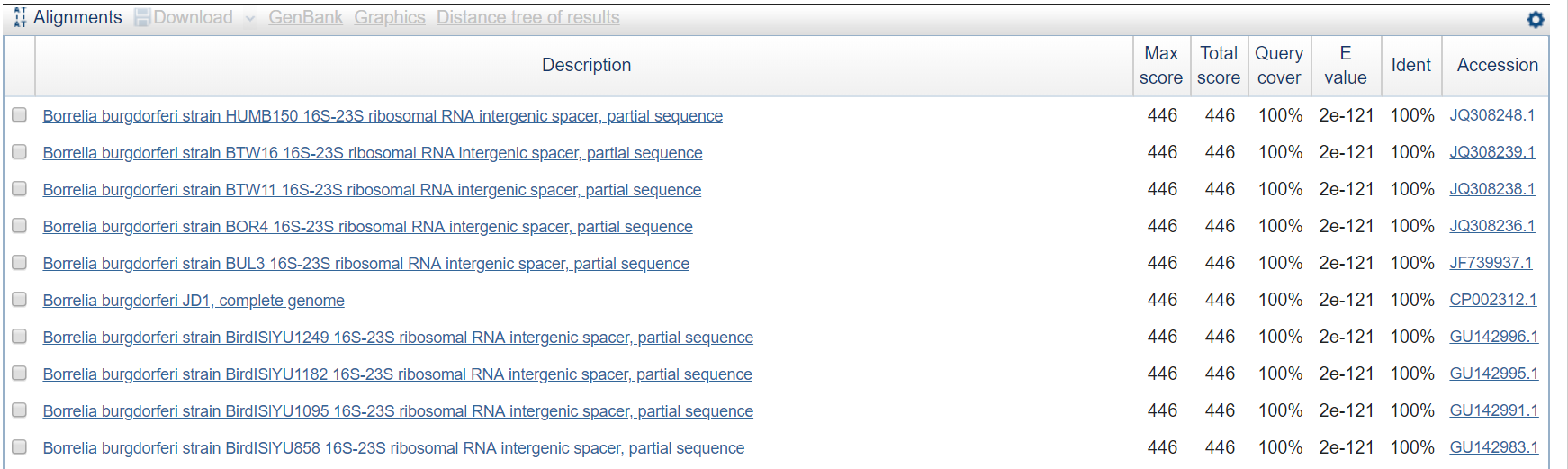 |
| C005_2016 | MH796084 | 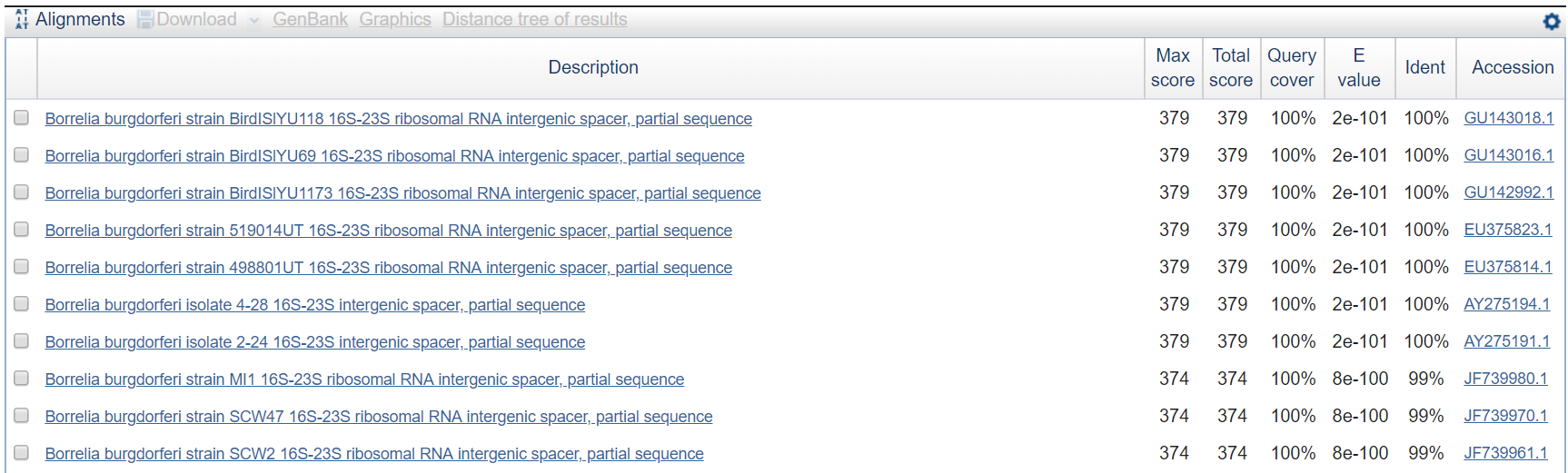 |
| C006_2016 | MH796085 | 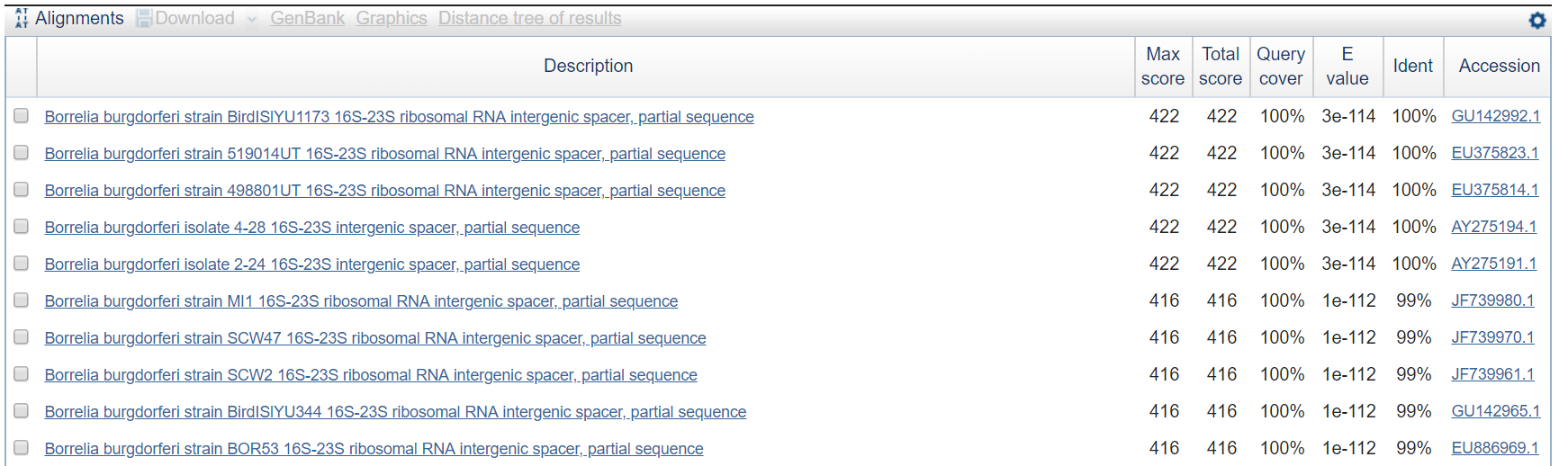 |
| C008_2016 | MH796086 | 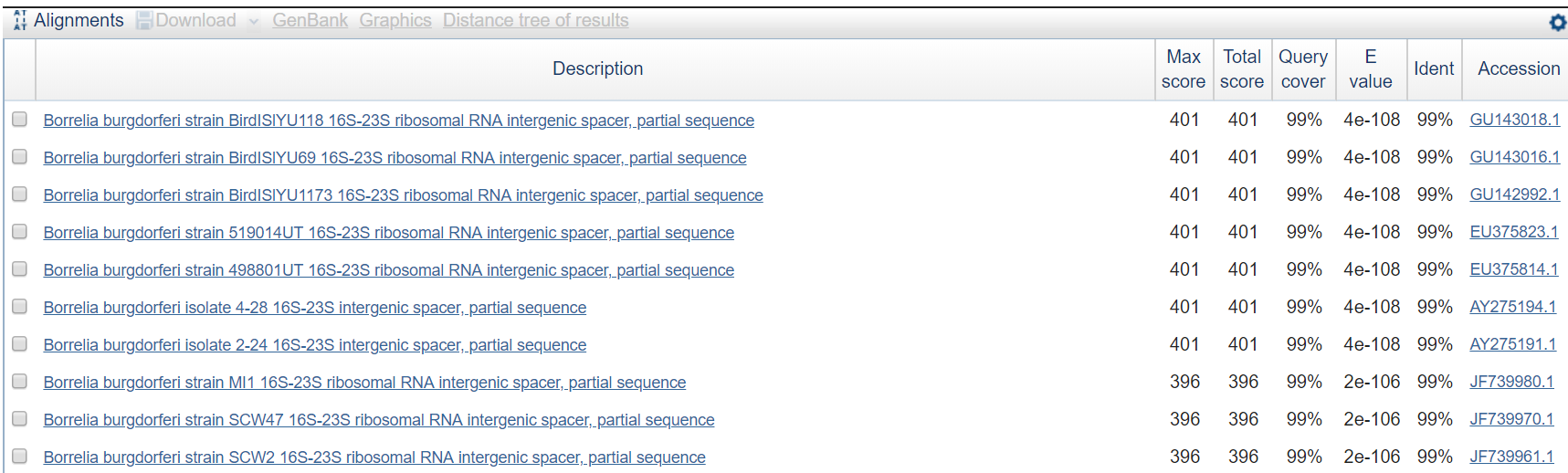 |
| C143_2016 | MH796087 | 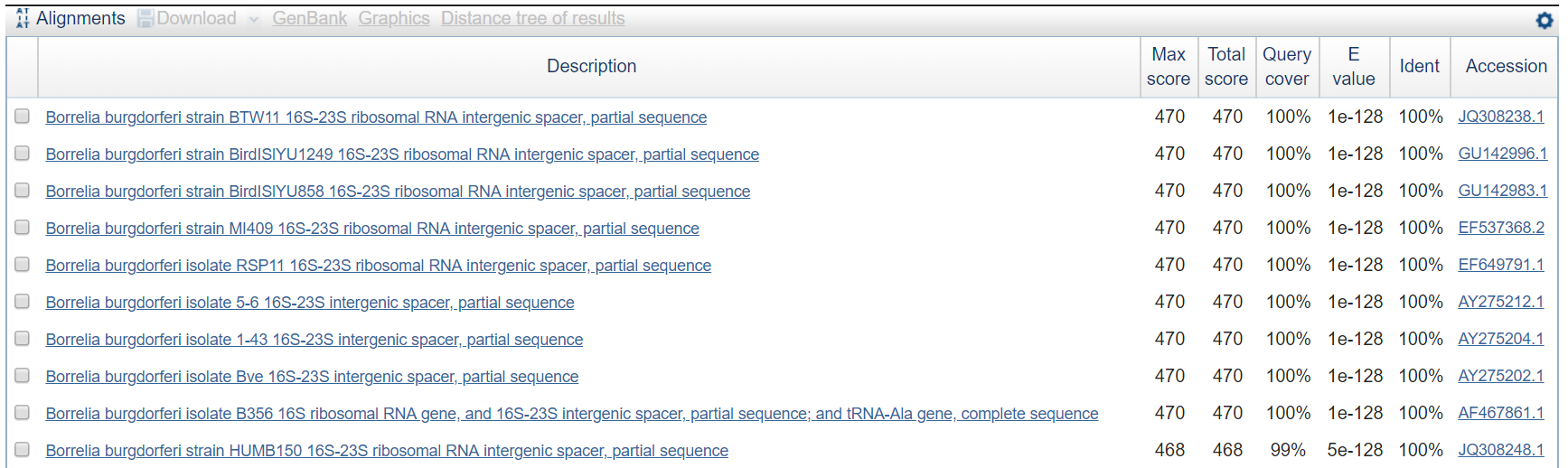 |
| C144_2016 | MH796088 | 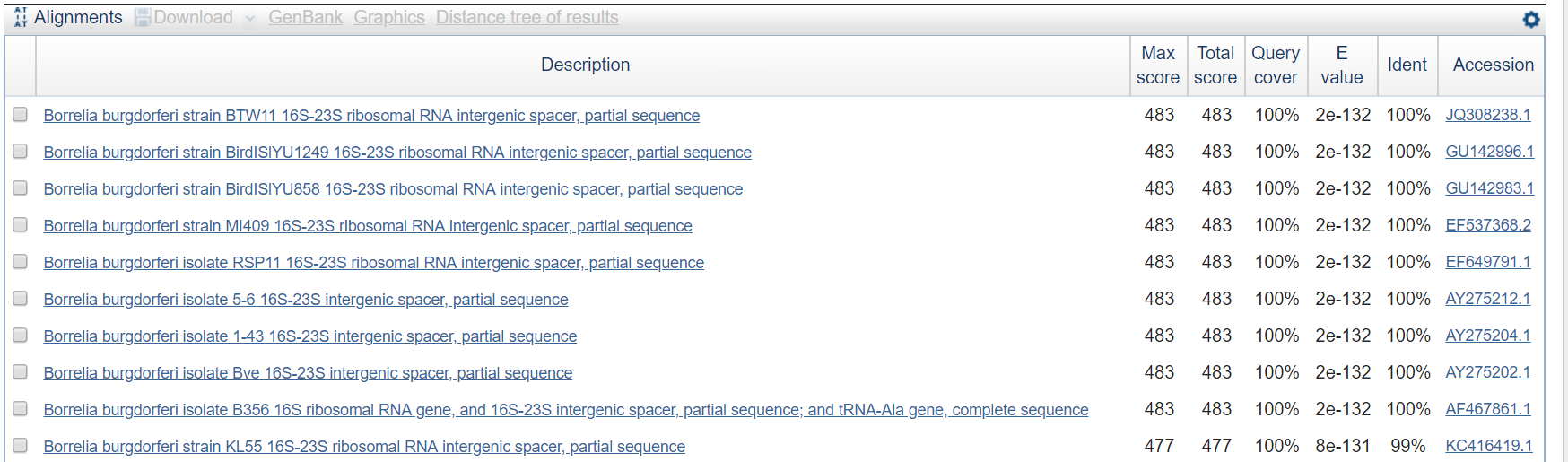 |
| C147_2016 | MH796089 | 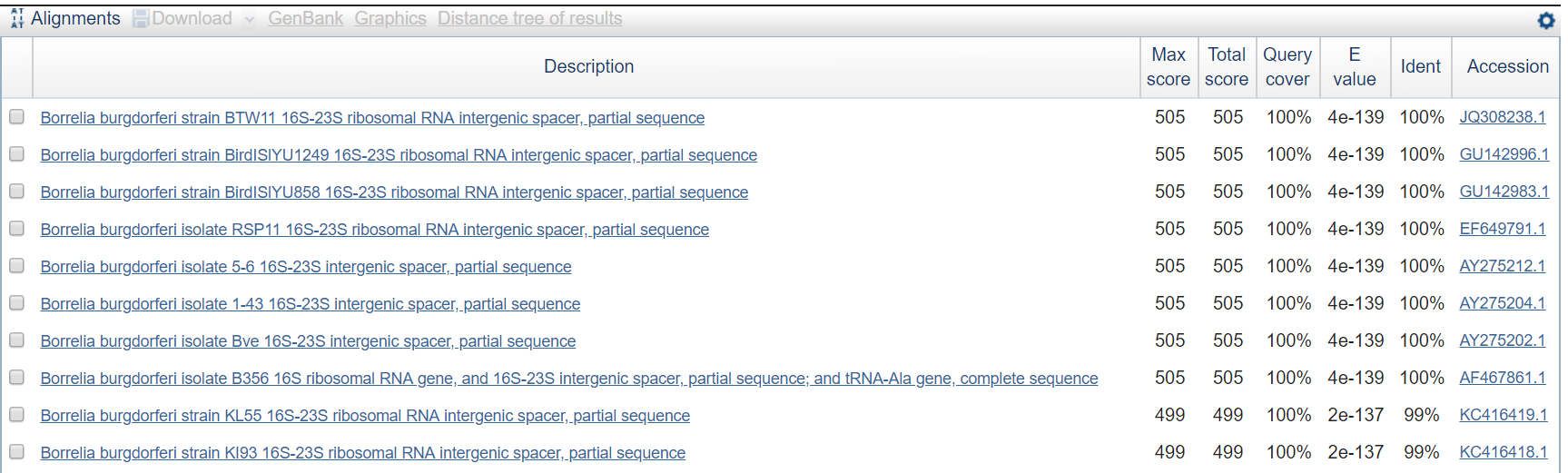 |
| C203_2016 | MH796090 | 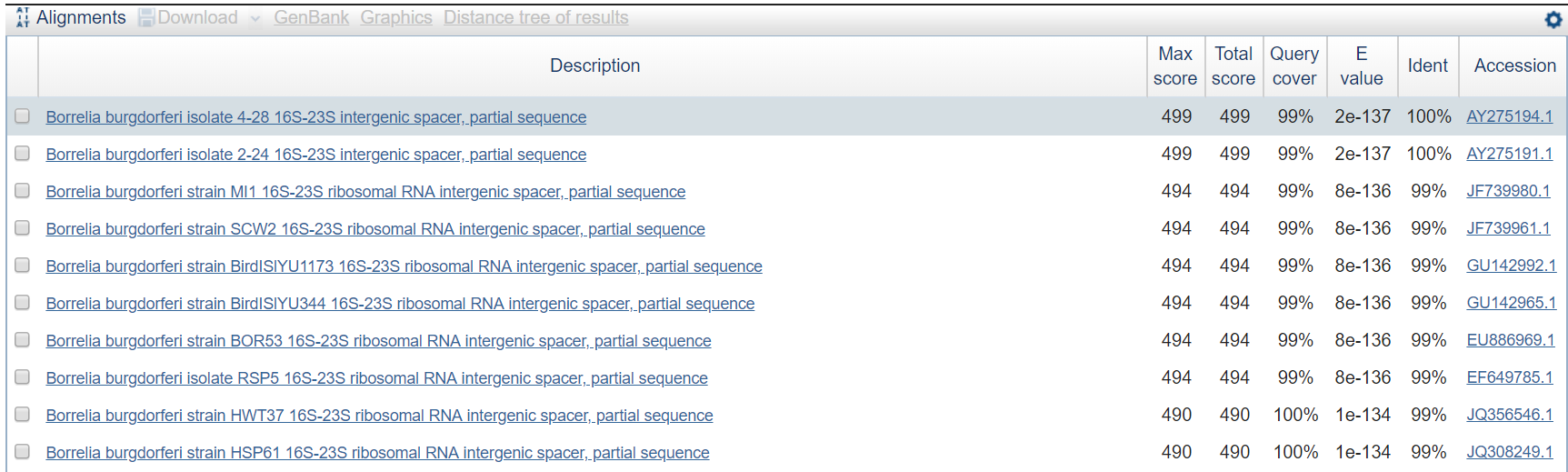 |
| C204_2016 | MH796091 | 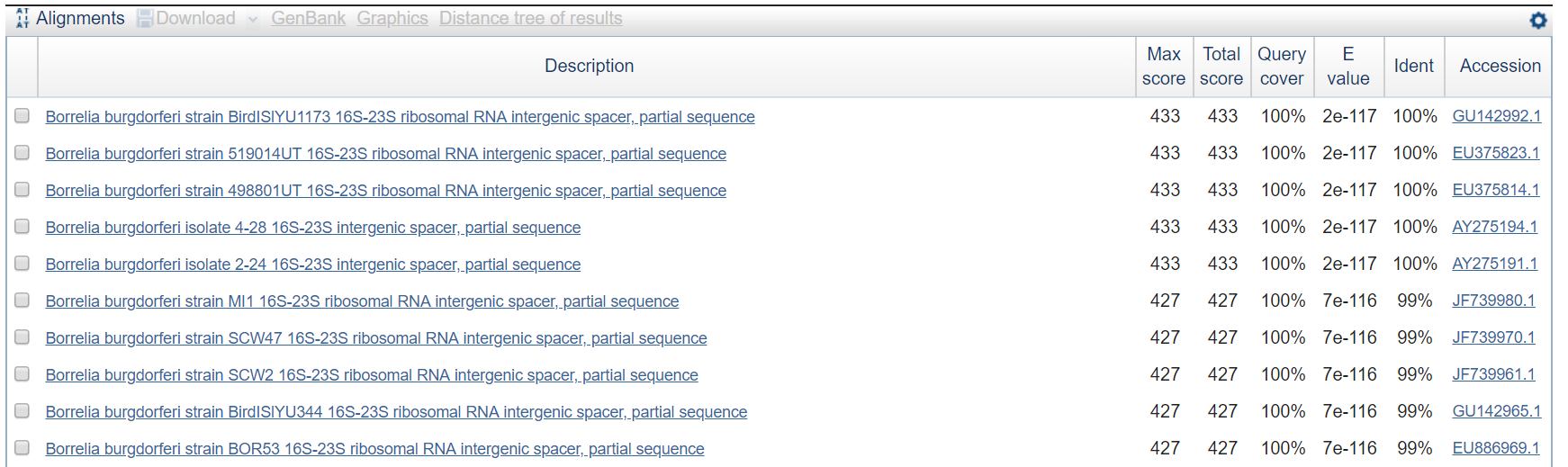 |
| C206_2016 | MH796092 | 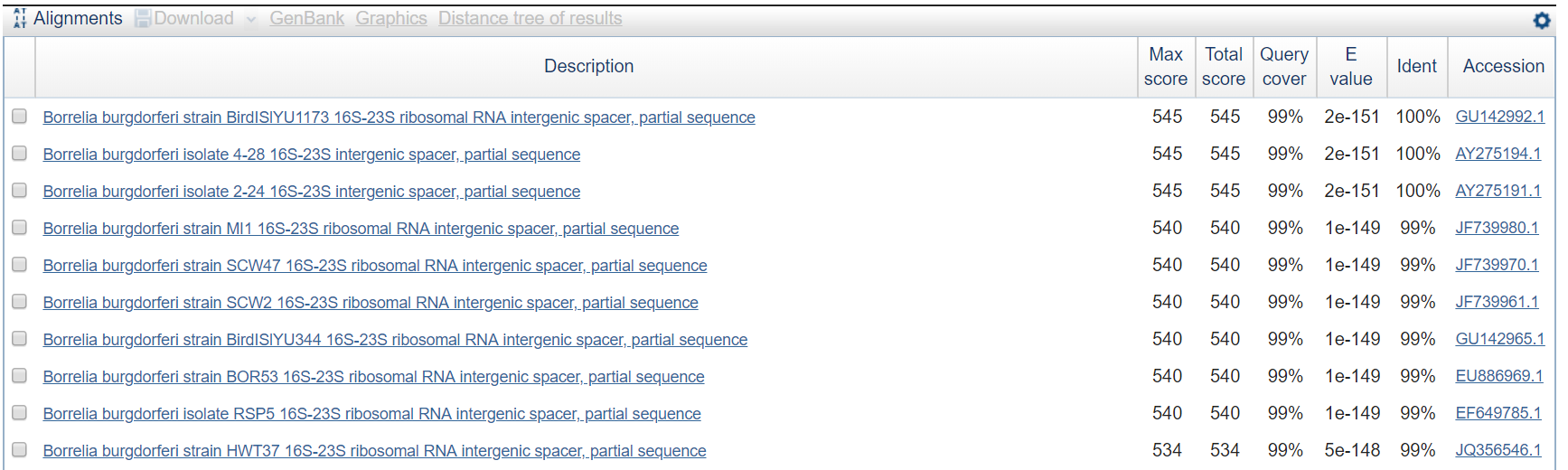 |
| C050_2017 | MH796093 | 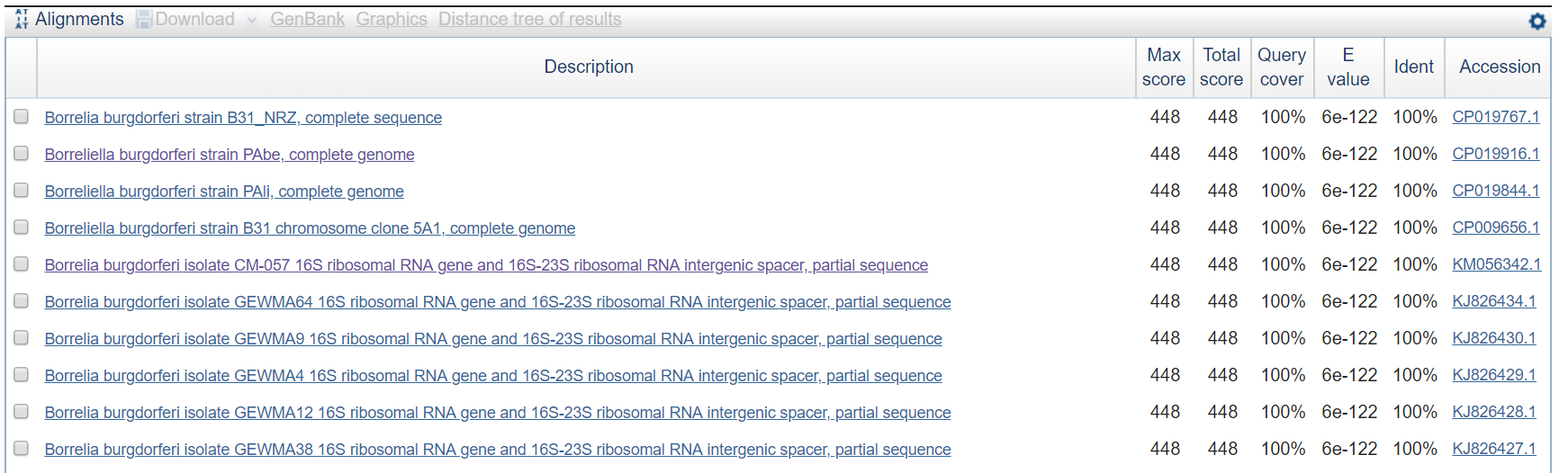 |
| C165_2016 | MH796094 | 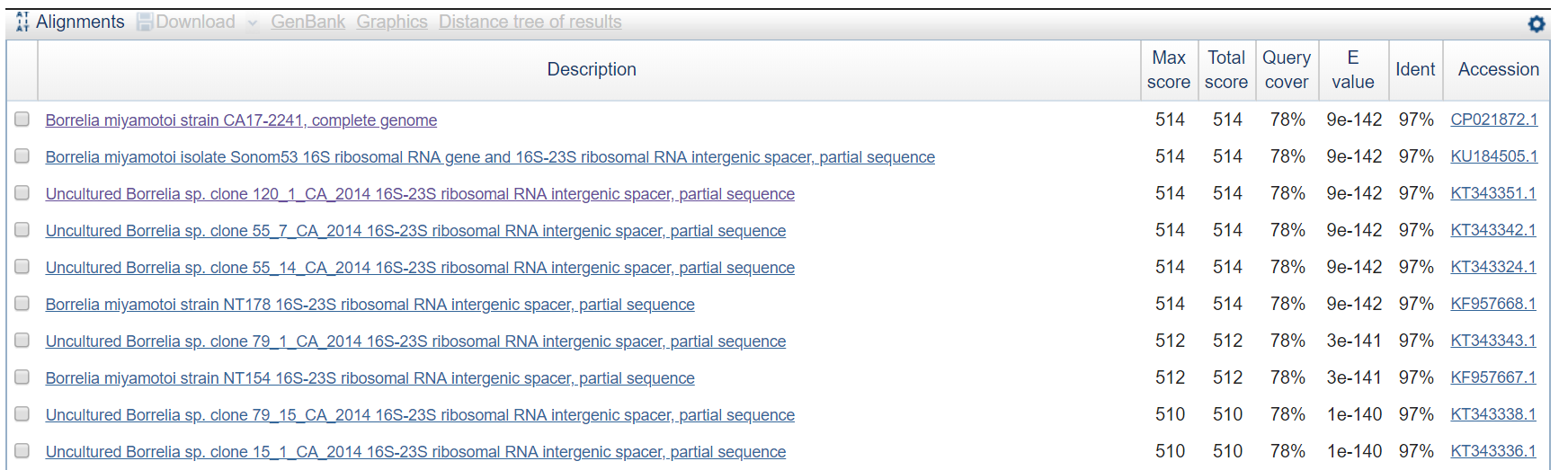 |
| C165_2016 | MH796095 | 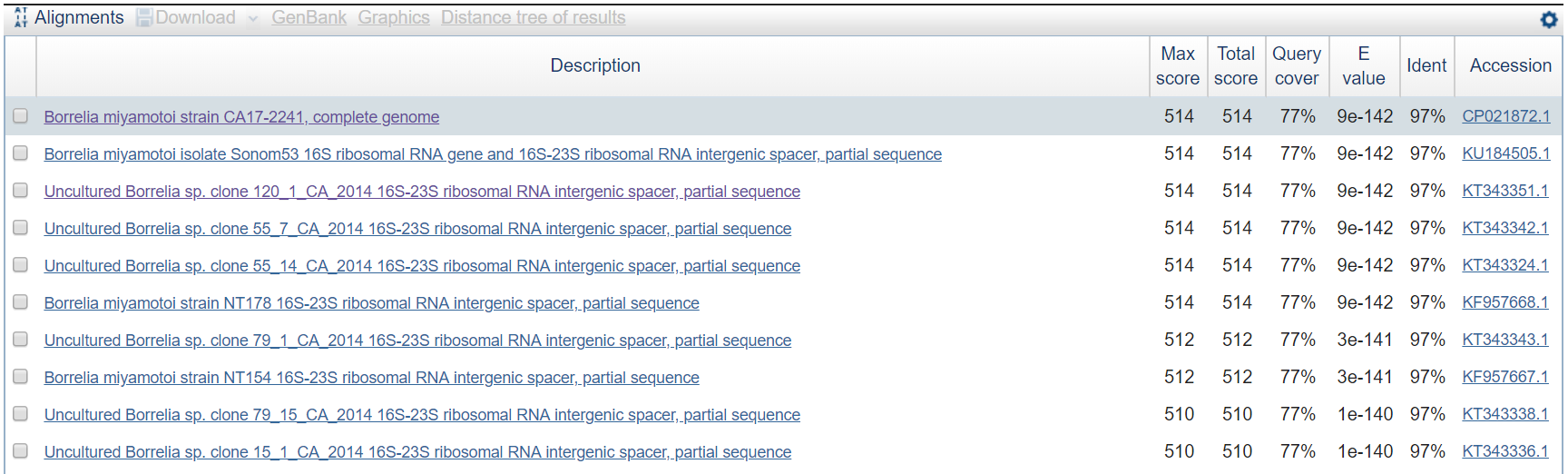 |
| C165_2016 | MH796096 | 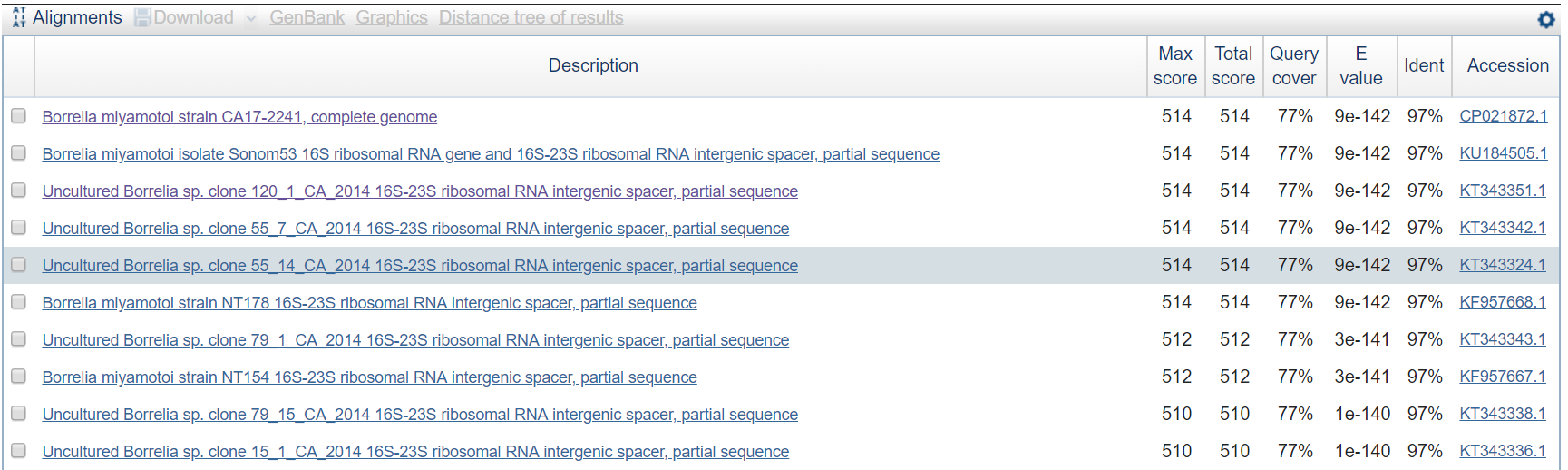 |
| C165_2016 | MH796097 | 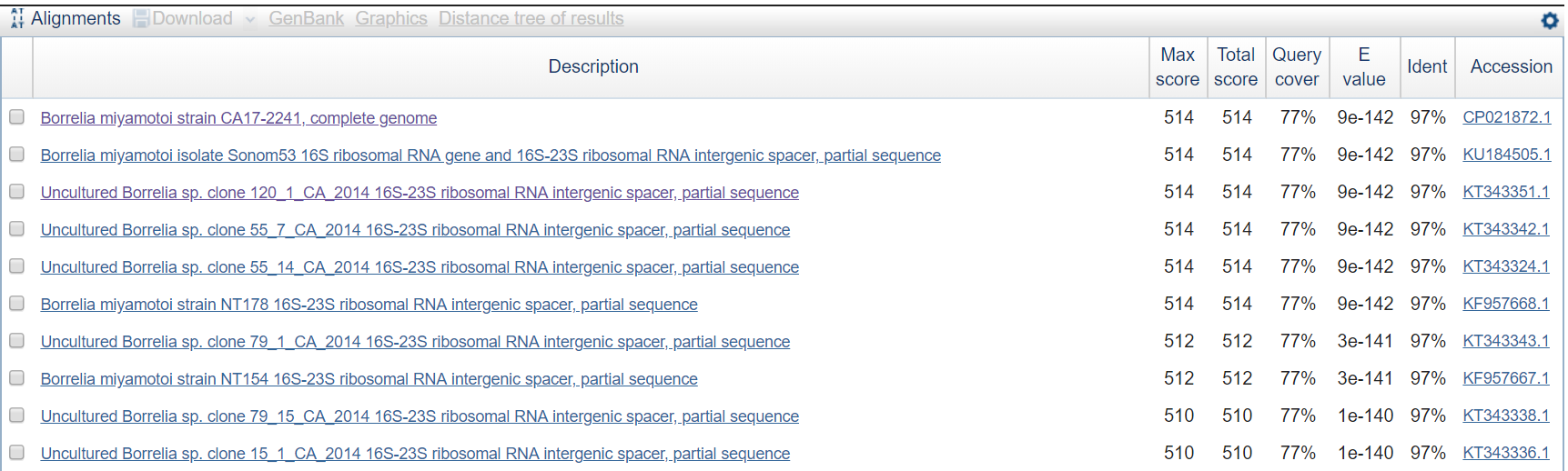 |
| C006_2016 | MH796098 | 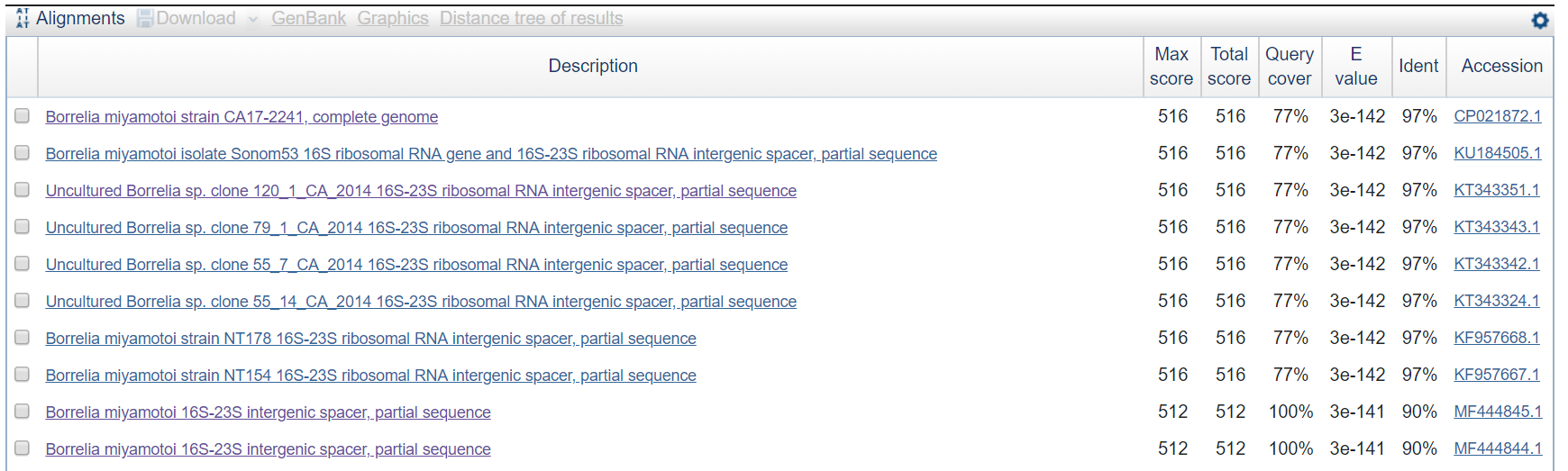 |
| C006_Fetus_2016 | MH796099 | 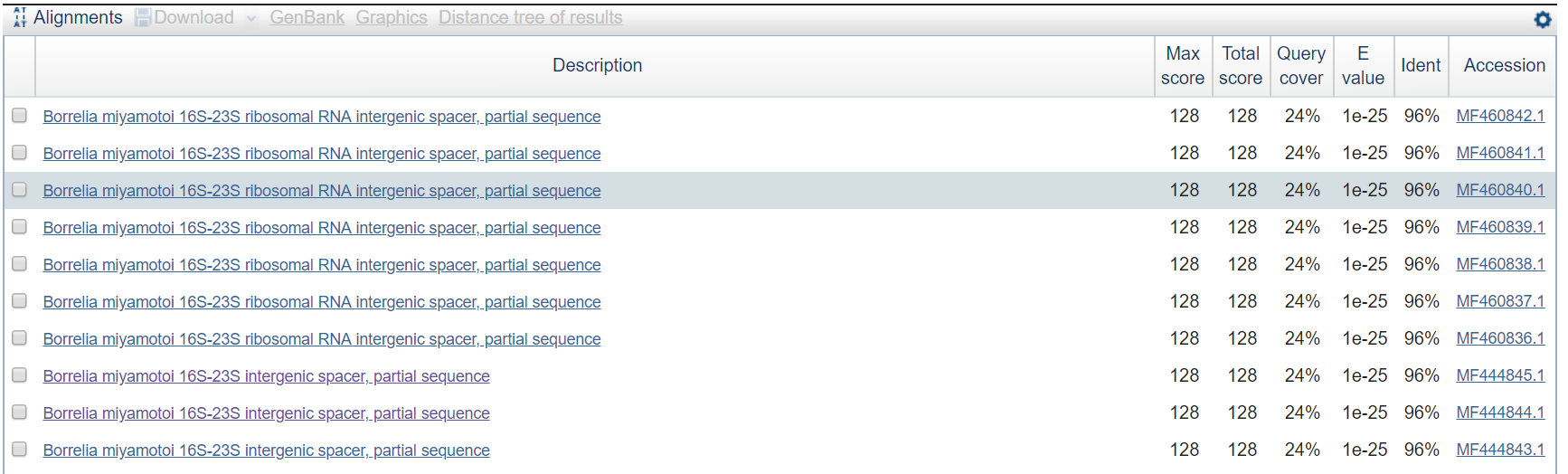 |
| C006_Fetus_2016 | MH796100 | 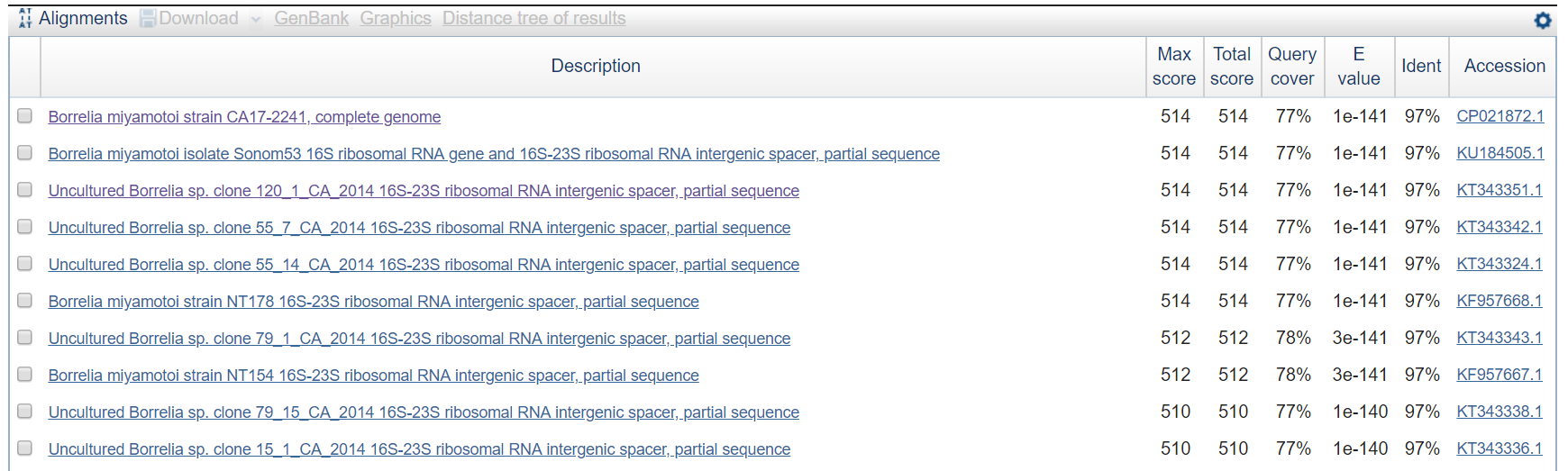 |
| C006_2016 | MH796101 | 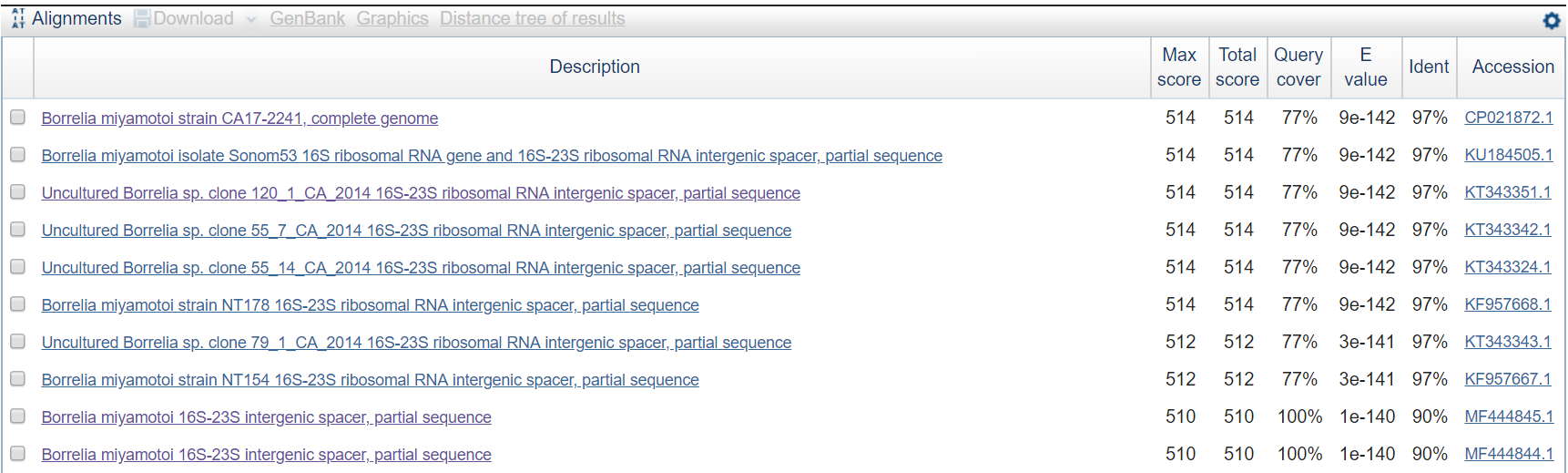 |
| C006_2016 | MH796102 | 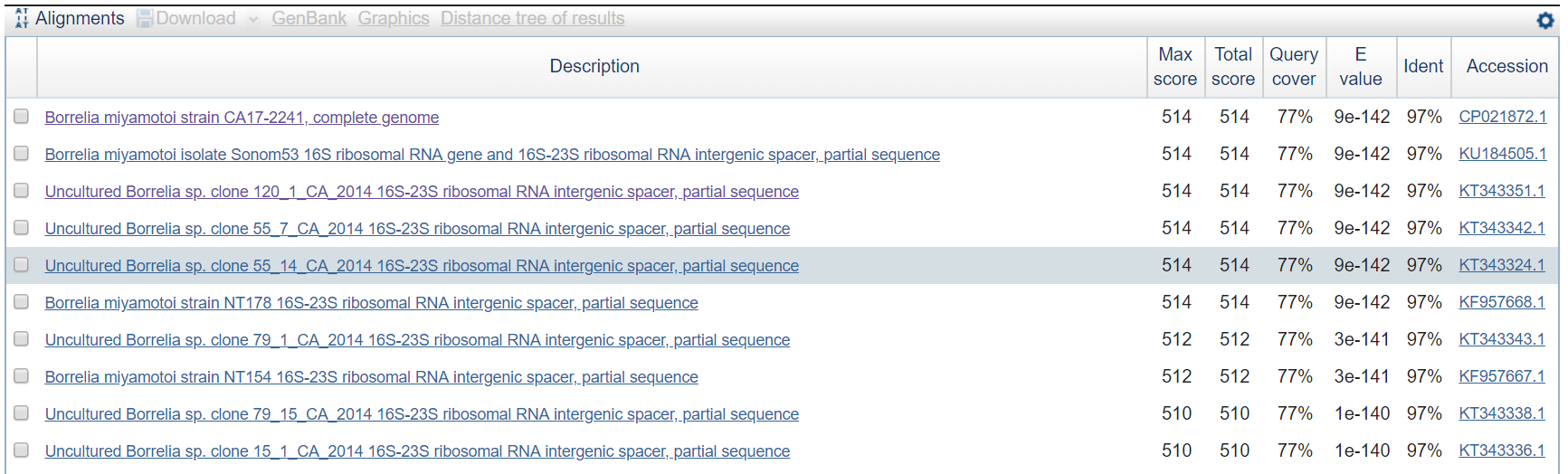 |
| C006_Fetus_2016 | MH796103 | No significant similarities. |
| C006_2016 | MH796104 | 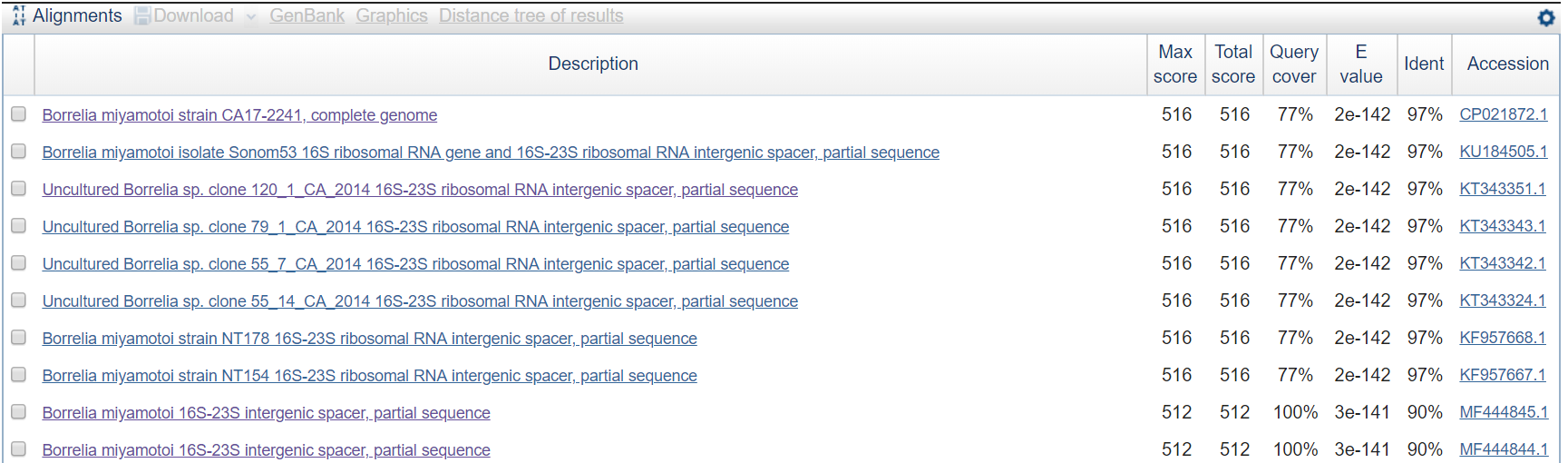 |
| C006_2016 | MH796105 | 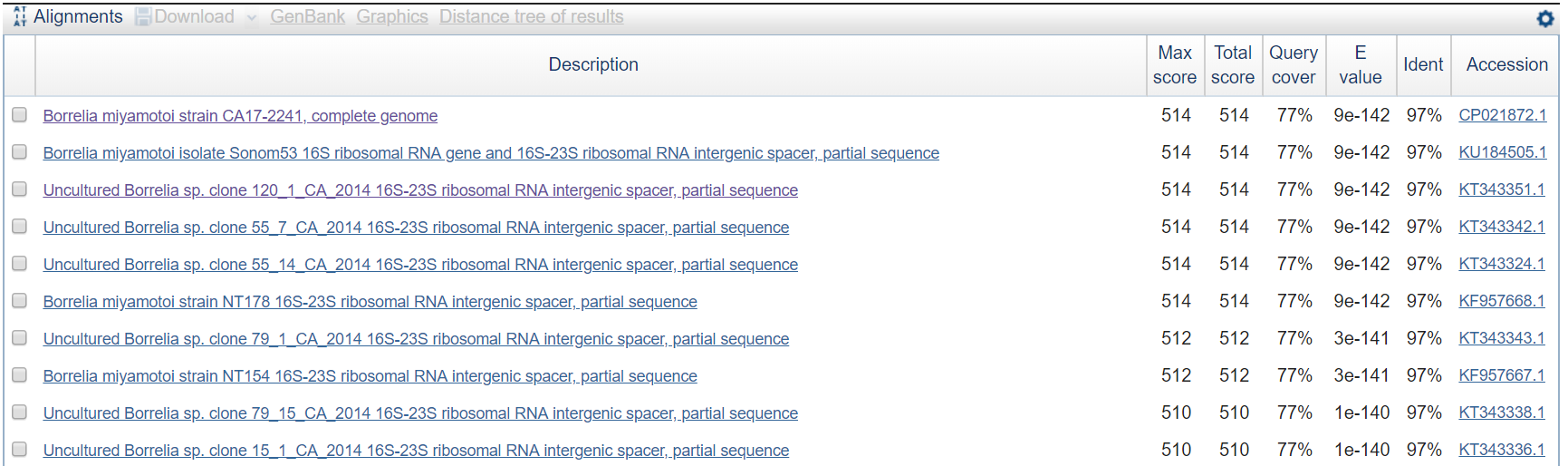 |
| C006_2016 | MH796106 | 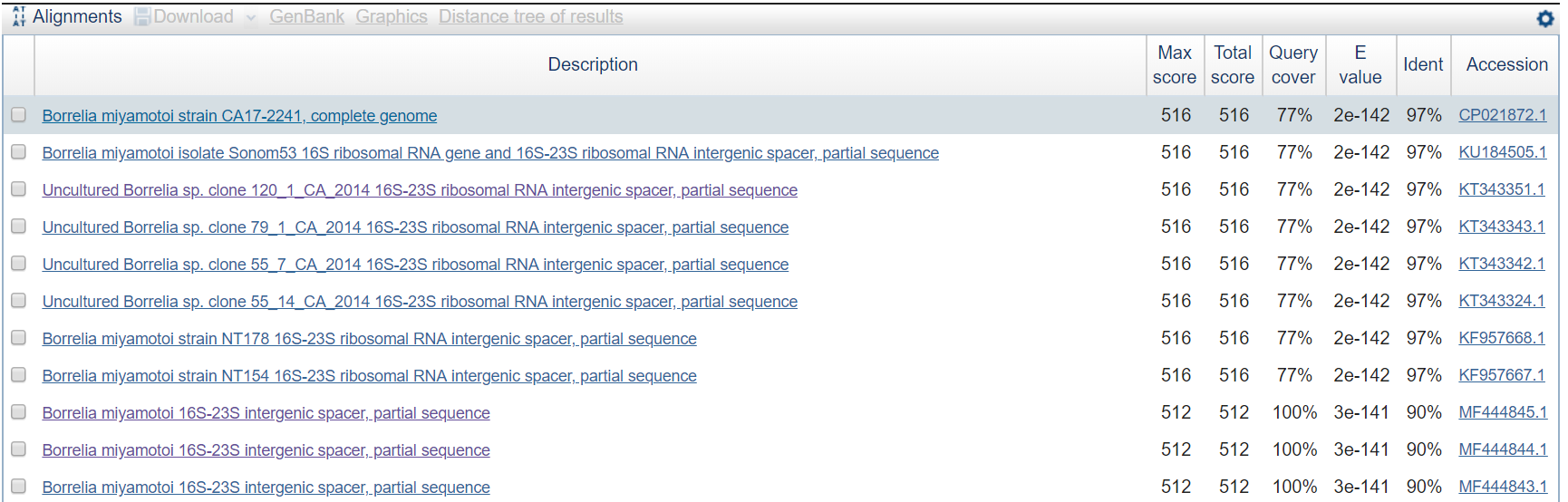 |
| C006_2016 | MH796107 | 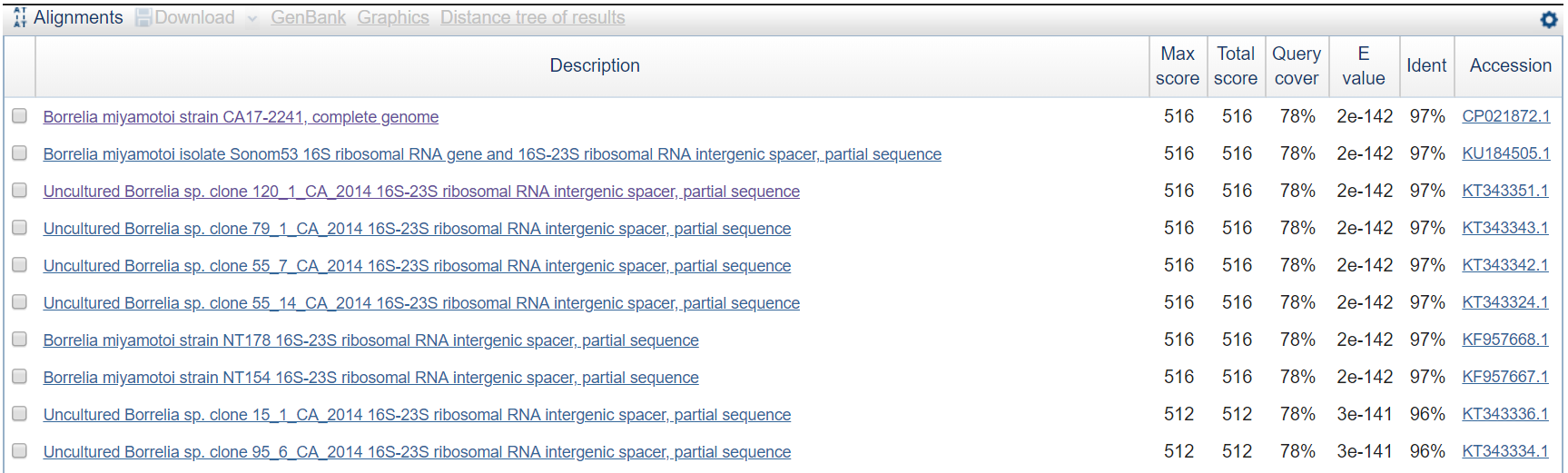 |
| C006_2016 | MH796108 | 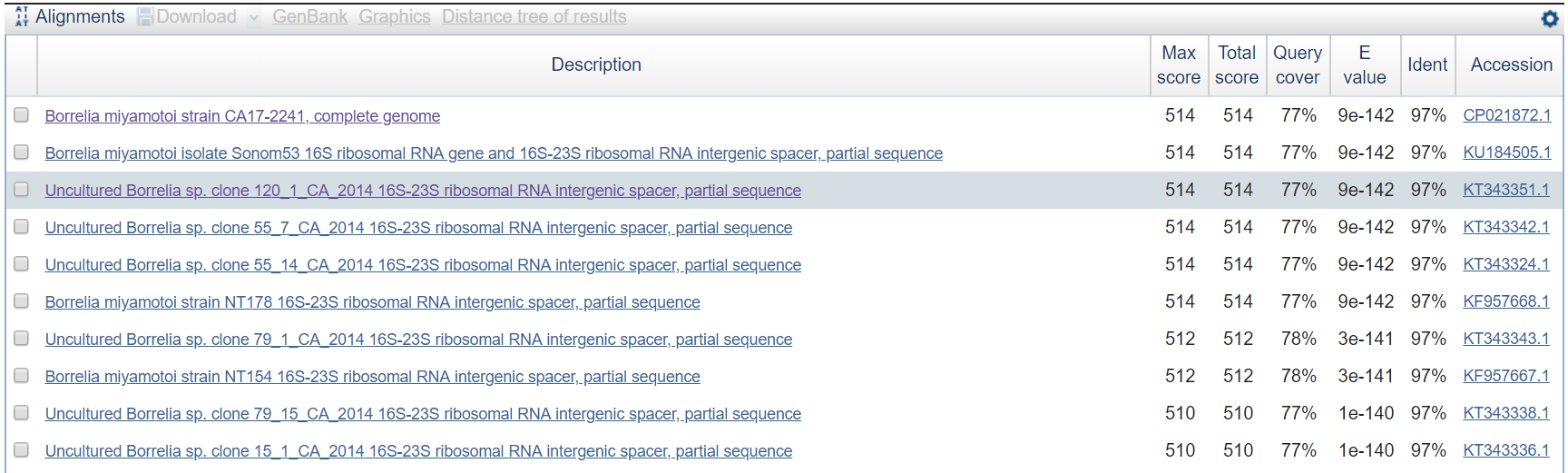 |
| C006_2016 | MH796109 | 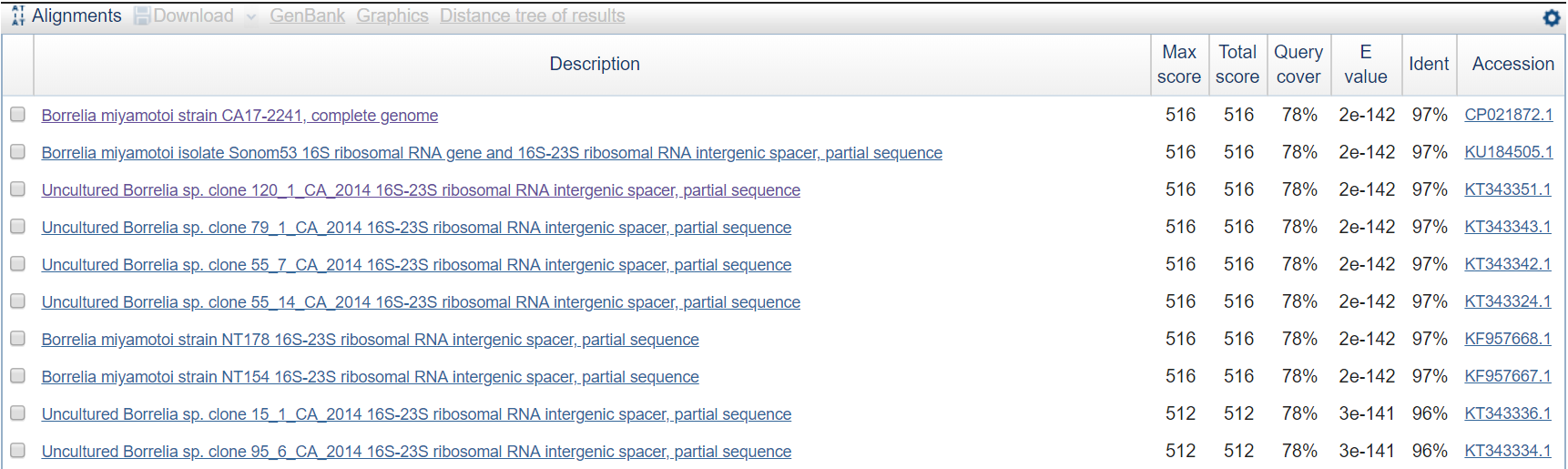 |
| C006_2016 | MH796110 | 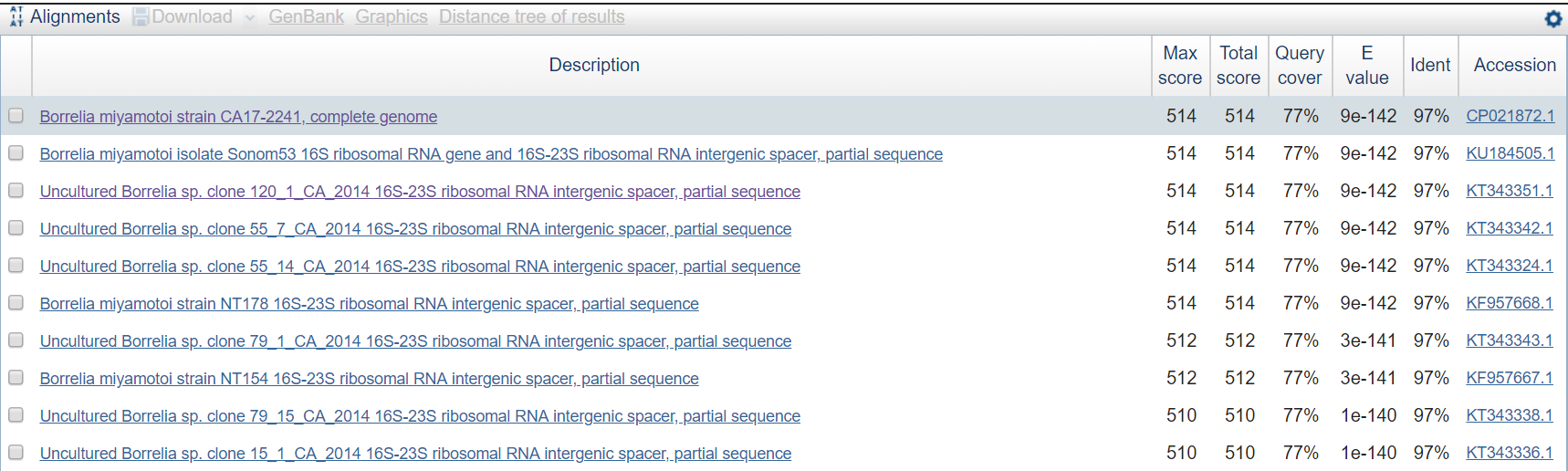 |
| C006_2016 | MH796111 | 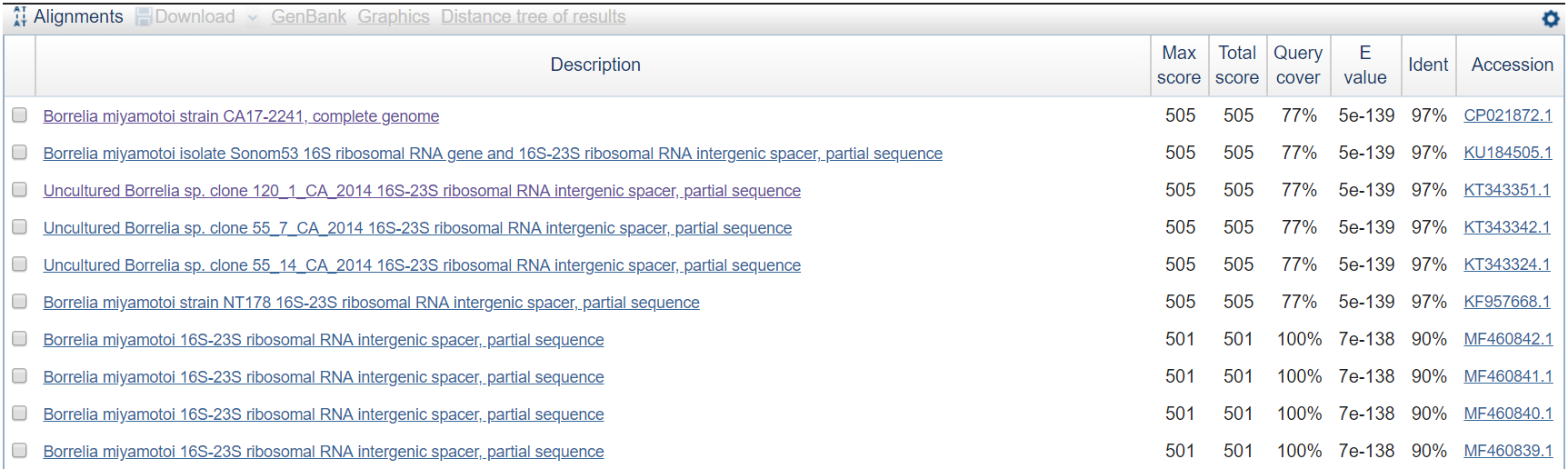 |
| C006_2016 | MH796112 | 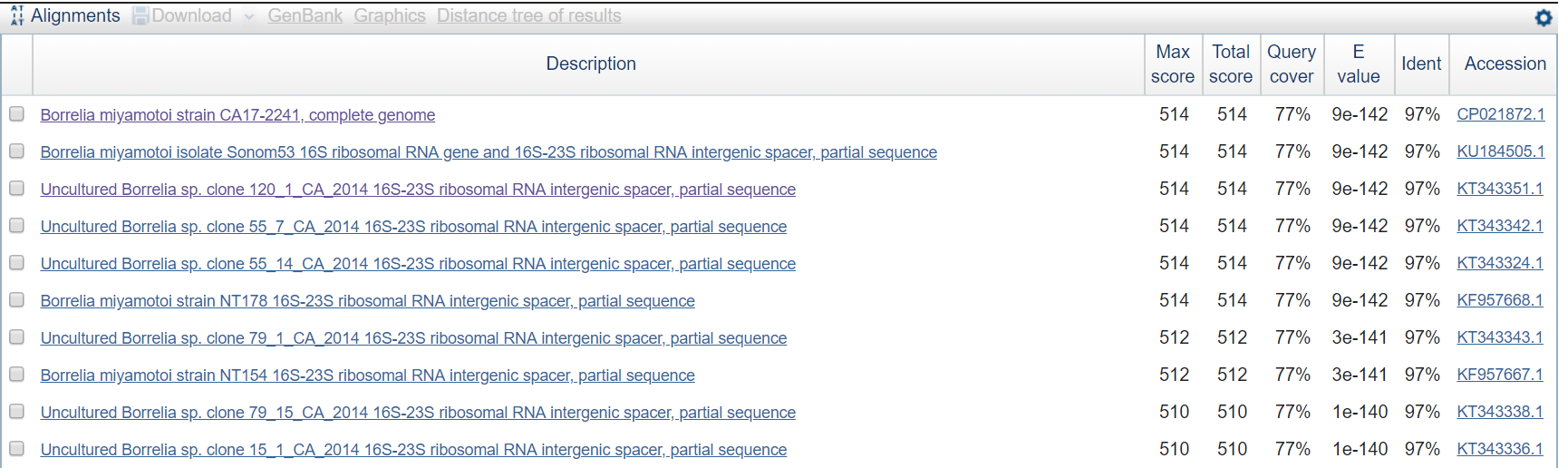 |
| C062_2017 | MH796113 | 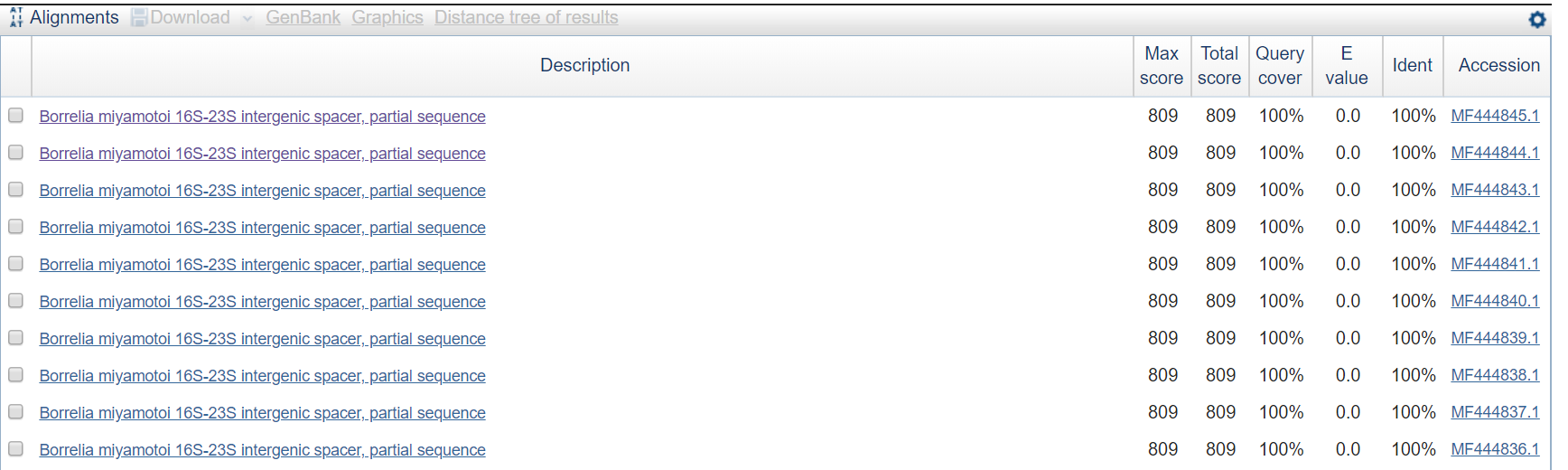 |
| C063_2017 | MH796114 | 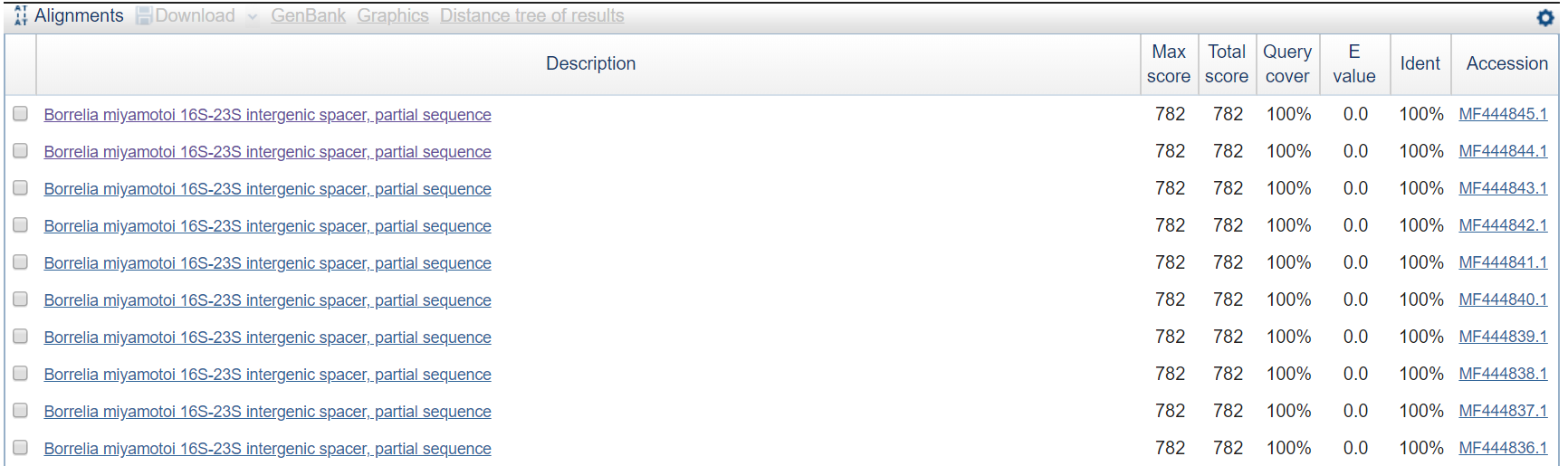 |
| C068_2017 | MH796115 | 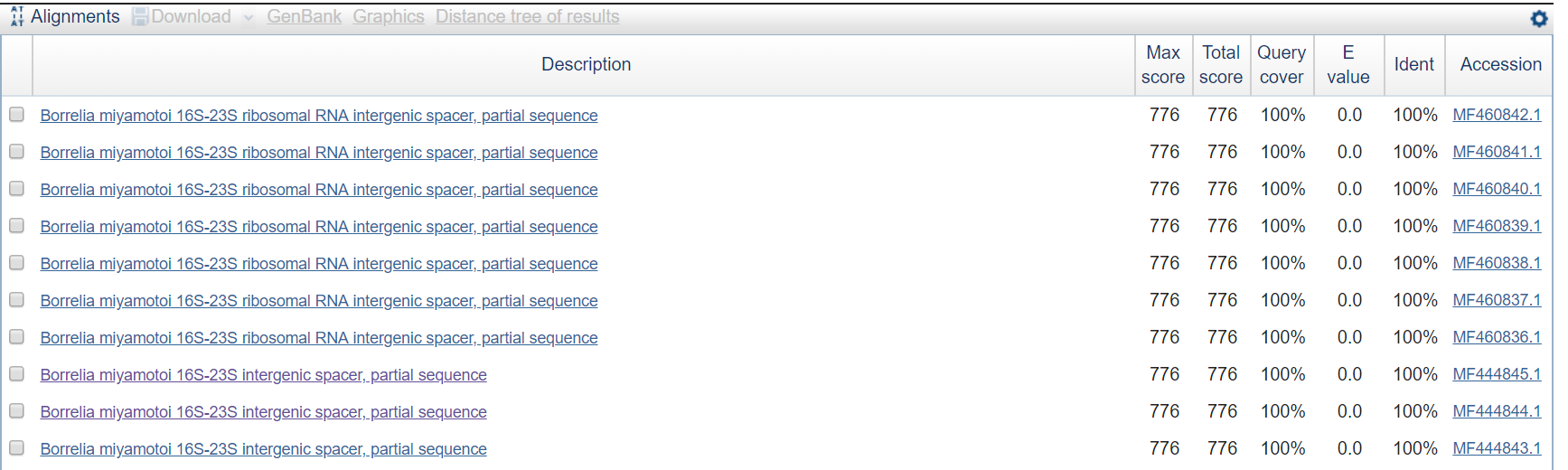 |
| C072_2017 | MH796116 | 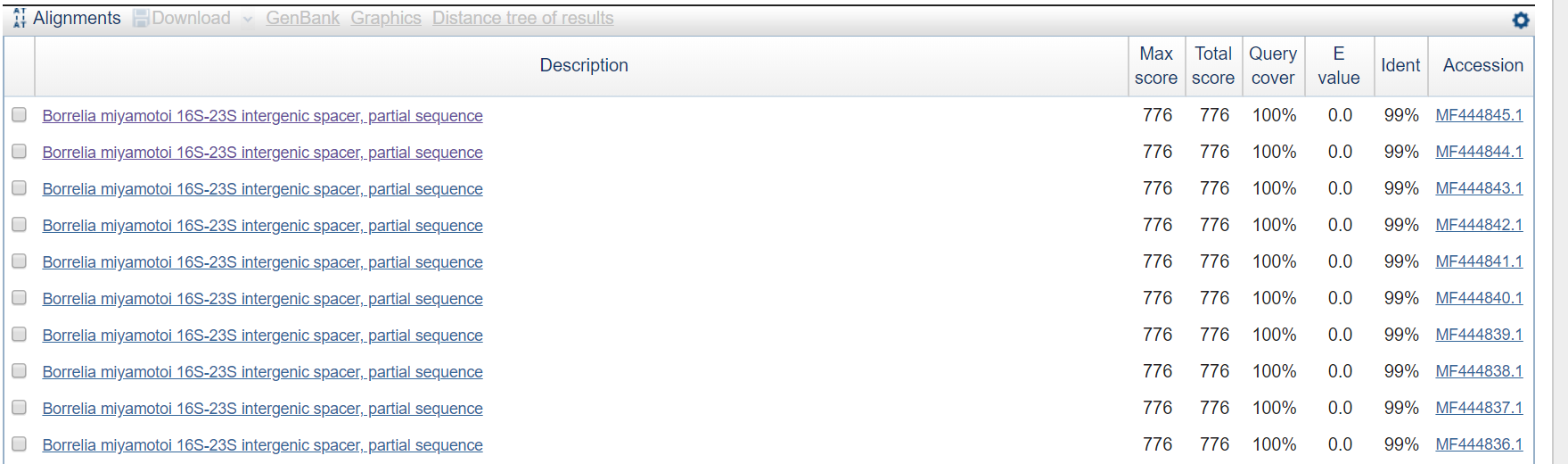 |
| C064_2017 | MH796117 | 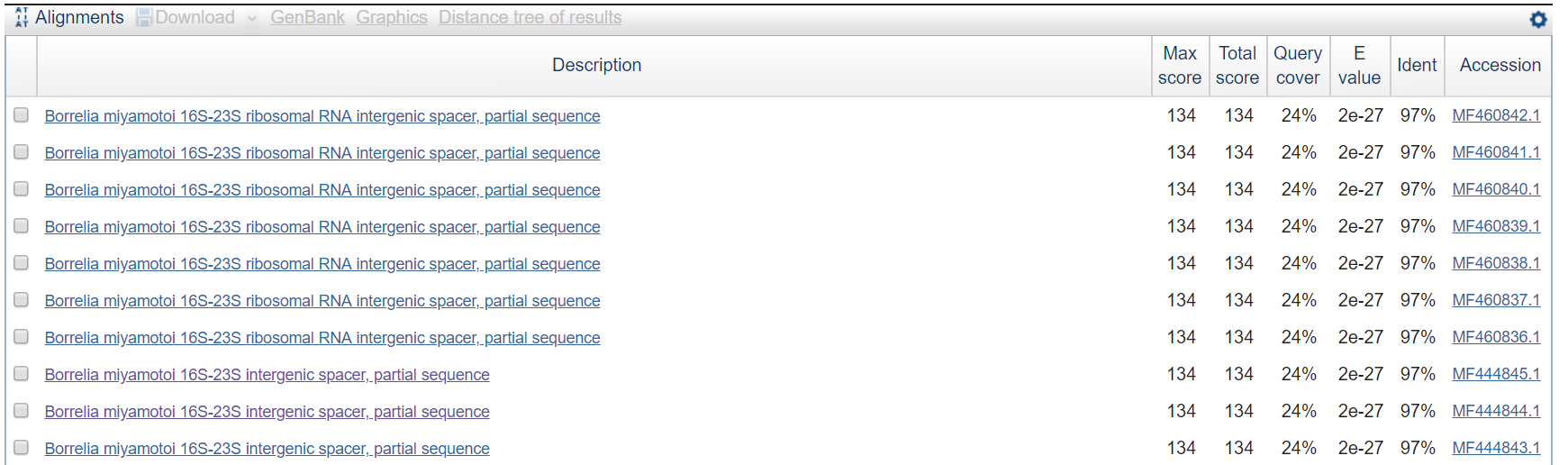 |
