# Supplementary material for: Borrelia burgdorferi and Borrelia miyamotoi in Atlantic Canadian wildlife
Source: PLoS One. 2022 Jan 21;17(1):e0262229. doi: 10.1371/journal.pone.0262229 (PMC8782396; doi:10.1371/journal.pone.0262229)
Supplement: S2 Table — Roadkill, denoted with an “R” sample ID, have their UTM coordinates (zone 20N) reported. The donated samples, denoted with a “C” sample ID, have their community or county of origin listed to protect the privacy of donors. The infection status of each animal is indicated by a 1, meaning detection of that Borrelia species, or 0 indicating it was negative. (DOCX) [file pone.0262229.s004.docx]

**S2 Table. Location data for all samples.** Roadkill, denoted with an “R” sample ID, have their UTM coordinates (zone 20N) reported. The donated samples, denoted with a “C” sample ID, have their community or county of origin listed to protect the privacy of donors. The infection status of each animal is indicated by a 1, meaning detection of that *Borrelia* species, or 0 indicating it was negative.

| Year | Sample | Species | *B. burgdorferi* | *B. miyamotoi* | County or Community | Province | Easting | Northing |
| --- | --- | --- | --- | --- | --- | --- | --- | --- |
| 2016 | R001 | Porcupine | 1 | 0 |  | NB | 388475 | 5091993 |
| 2016 | R002 | Porcupine | 0 | 0 |  | NB | 385681 | 5093634 |
| 2016 | R003 | Porcupine | 0 | 0 |  | NB | 389883 | 5090977 |
| 2016 | R004 | American crow | 0 | 0 |  | NB | 393396 | 5085029 |
| 2016 | R005 | Porcupine | 0 | 0 |  | NB | 388629 | 5080787 |
| 2016 | R006 | American crow | 1 | 0 |  | NB | 392010 | 5086194 |
| 2016 | R007 | Porcupine | 0 | 0 |  | NB | 392010 | 5086194 |
| 2016 | R008 | Common garter snake | 0 | 0 |  | NB | 401668 | 5083087 |
| 2016 | R009 | American crow | 0 | 0 |  | NB | 390408 | 5090358 |
| 2016 | R010 | Snow shoe hare | 0 | 0 |  | NB | 388196 | 5092167 |
| 2016 | R011 | Raccoon | 0 | 0 |  | NB | 381069 | 5098509 |
| 2016 | R012 | Porcupine | 0 | 0 |  | NB | 376207 | 5106017 |
| 2016 | R013 | Porcupine | 0 | 0 |  | NB | 372246 | 5109758 |
| 2016 | R014 | American crow | 0 | 0 |  | NB | 406612 | 5088400 |
| 2016 | R015 | Mallard | 0 | 0 |  | NB | 392851 | 5085337 |
| 2016 | R016 | Groundhog | 0 | 0 |  | NB | 376626 | 5105205 |
| 2016 | R017 | Porcupine | 0 | 0 |  | NB | 373464 | 5109206 |
| 2016 | R018 | Porcupine | 0 | 0 |  | NB | 377124 | 5102081 |
| 2016 | R019 | Porcupine | 0 | 0 |  | NB | 384381 | 5094265 |
| 2016 | R020 | Porcupine | 0 | 0 |  | NB | 389907 | 5090879 |
| 2016 | R021 | Raccoon | 0 | 0 |  | NB | 391163 | 5087855 |
| 2016 | R022 | American robin | 0 | 0 |  | NB | 387840 | 5080872 |
| 2016 | R023 | Ruffed grouse | 0 | 0 |  | NB | 394430 | 5084371 |
| 2016 | R024 | Raccoon | 0 | 0 |  | NB | 395245 | 5083702 |
| 2016 | R025 | American crow | 0 | 0 |  | NB | 400214 | 5081548 |
| 2016 | R026 | Groundhog | 0 | 0 |  | NB | 402598 | 5082248 |
| 2016 | R027 | Porcupine | 1 | 0 |  | NB | 390742 | 5113249 |
| 2016 | R028 | Snow shoe hare | 0 | 0 |  | NB | 386074 | 5093386 |
| 2016 | R029 | Rock pigeon | 0 | 0 |  | NB | 393598 | 5083821 |
| 2016 | R030 | Porcupine | 0 | 0 |  | NB | 391822 | 5086403 |
| 2016 | R031 | Porcupine | 0 | 0 |  | NB | 371742 | 5109804 |
| 2016 | R032 | Porcupine | 0 | 0 |  | NB | 391462 | 5086945 |
| 2016 | R033 | Raccoon | 0 | 0 |  | NB | 388969 | 5091607 |
| 2016 | R034 | American crow | 0 | 0 |  | NB | 391172 | 5088102 |
| 2016 | R035 | Red Squirrel | 0 | 0 |  | NB | 393330 | 5083824 |
| 2016 | R036 | Porcupine | 0 | 0 |  | NB | 386262 | 5093247 |
| 2016 | R037 | Porcupine | 0 | 0 |  | NB | 413188 | 5095039 |
| 2016 | R038 | American crow | 0 | 0 |  | NB | 394124 | 5084602 |
| 2016 | R039 | Raccoon | 0 | 0 |  | NB | 391115 | 5089448 |
| 2016 | R040 | Raccoon | 0 | 0 |  | NB | 387284 | 5092676 |
| 2016 | R041 | Porcupine | 0 | 0 |  | NB | 385021 | 5093894 |
| 2016 | R042 | American crow | 0 | 0 |  | NB | 390610 | 5089988 |
| 2016 | R043 | Snow shoe hare | 0 | 0 |  | NB | 379977 | 5092883 |
| 2016 | R044 | Red fox | 0 | 0 |  | NB | 399060 | 5082011 |
| 2016 | R045 | Raccoon | 0 | 0 |  | NB | 404589 | 5085218 |
| 2016 | R046 | Rock pigeon | 0 | 0 |  | NB | 379833 | 5099814 |
| 2016 | R047 | Ruffed grouse | 0 | 0 |  | NB | 407151 | 5089194 |
| 2016 | R048 | American crow | 0 | 0 |  | NB | 341920 | 5099759 |
| 2016 | R049 | Porcupine | 0 | 0 |  | NB | 381463 | 5097448 |
| 2016 | R050 | American crow | 0 | 0 |  | NB | 379977 | 5092883 |
| 2016 | R051 | American crow | 0 | 0 |  | NB | 379977 | 5092883 |
| 2016 | R052 | Porcupine | 0 | 0 |  | NB | 382169 | 5095774 |
| 2016 | R053 | Groundhog | 0 | 0 |  | NB | 391257 | 5087625 |
| 2016 | R054 | American black duck | 0 | 0 |  | NB | 413585 | 5096214 |
| 2016 | R055 | Porcupine | 0 | 0 |  | NB | 384761 | 5094148 |
| 2016 | R056 | Muskrat | 0 | 0 |  | NB | 413549 | 5096442 |
| 2016 | R057 | Raccoon | 0 | 0 |  | NB | 221758 | 5098374 |
| 2016 | R058 | Muskrat | 0 | 0 |  | NB | 221758 | 5098374 |
| 2016 | C001 | Red Squirrel | 0 | 0 | Sackville | NB |  |  |
| 2016 | C002 | Deer mouse | 0 | 0 | Sackville | NB |  |  |
| 2016 | C003 | Meadow vole | 0 | 0 | Aulac | NB |  |  |
| 2016 | C004 | Meadow vole | 0 | 0 | Aulac | NB |  |  |
| 2016 | C005 | Meadow vole | 1 | 0 | Aulac | NB |  |  |
| 2016 | C006 | Jumping mouse | 1 | 1 | Aulac | NB |  |  |
| 2016 | C007 | Meadow vole | 0 | 0 | Aulac | NB |  |  |
| 2016 | C008 | Jumping mouse | 1 | 0 | Jolicure | NB |  |  |
| 2016 | C009 | Meadow vole | 0 | 0 | Jolicure | NB |  |  |
| 2016 | C010 | Brown rat | 0 | 0 | Jolicure | NB |  |  |
| 2016 | C011 | Brown rat | 0 | 0 | Jolicure | NB |  |  |
| 2016 | C012 | Brown rat | 0 | 0 | Jolicure | NB |  |  |
| 2016 | C013 | Meadow vole | 0 | 0 | Jolicure | NB |  |  |
| 2016 | C014 | Brown rat | 0 | 0 | Jolicure | NB |  |  |
| 2016 | C015 | Brown rat | 0 | 0 | Jolicure | NB |  |  |
| 2016 | C016 | Meadow vole | 0 | 0 | Jolicure | NB |  |  |
| 2016 | C017 | Deer mouse | 0 | 0 | Jolicure | NB |  |  |
| 2016 | C018 | Deer mouse | 0 | 0 | Jolicure | NB |  |  |
| 2016 | C019 | Deer mouse | 0 | 0 | Jolicure | NB |  |  |
| 2016 | C020 | Deer mouse | 0 | 0 | Jolicure | NB |  |  |
| 2016 | C021 | Deer mouse | 0 | 0 | Jolicure | NB |  |  |
| 2016 | C022 | Deer mouse | 0 | 0 | Jolicure | NB |  |  |
| 2016 | C023 | Deer mouse | 0 | 0 | Jolicure | NB |  |  |
| 2016 | C024 | Deer mouse | 0 | 0 | Jolicure | NB |  |  |
| 2016 | C025 | Deer mouse | 0 | 0 | Jolicure | NB |  |  |
| 2016 | C026 | Deer mouse | 0 | 0 | Jolicure | NB |  |  |
| 2016 | C027 | Deer mouse | 0 | 0 | Jolicure | NB |  |  |
| 2016 | C028 | Deer mouse | 0 | 0 | Jolicure | NB |  |  |
| 2016 | C029 | Deer mouse | 0 | 0 | Jolicure | NB |  |  |
| 2016 | C030 | Deer mouse | 0 | 0 | Jolicure | NB |  |  |
| 2016 | C031 | Deer mouse | 0 | 0 | Jolicure | NB |  |  |
| 2016 | C032 | Deer mouse | 0 | 0 | Jolicure | NB |  |  |
| 2016 | C033 | Shrew | 0 | 0 | Jolicure | NB |  |  |
| 2016 | C034 | Jumping mouse | 0 | 0 | Aulac | NB |  |  |
| 2016 | C035 | Meadow vole | 0 | 0 | Aulac | NB |  |  |
| 2016 | C036 | Deer mouse | 0 | 0 | Sackville | NB |  |  |
| 2016 | C037 | Meadow vole | 0 | 0 | Sackville | NB |  |  |
| 2016 | C038 | Jumping mouse | 0 | 0 | Sackville | NB |  |  |
| 2016 | C039 | Red Squirrel | 0 | 0 | Sackville | NB |  |  |
| 2016 | C040 | Meadow vole | 0 | 0 | Aulac | NB |  |  |
| 2016 | C041 | Meadow vole | 0 | 0 | Aulac | NB |  |  |
| 2016 | C043 | Meadow vole | 0 | 0 | Aulac | NB |  |  |
| 2016 | C044 | Deer mouse | 0 | 0 | Aulac | NB |  |  |
| 2016 | C045 | Meadow vole | 0 | 0 | Sackville | NB |  |  |
| 2016 | C046 | Meadow vole | 0 | 0 | Sackville | NB |  |  |
| 2016 | C047 | Meadow vole | 0 | 0 | Sackville | NB |  |  |
| 2016 | C048 | Meadow vole | 0 | 0 | Sackville | NB |  |  |
| 2016 | C049 | Meadow vole | 0 | 0 | Sackville | NB |  |  |
| 2016 | C050 | Meadow vole | 0 | 0 | Sackville | NB |  |  |
| 2016 | C051 | Meadow vole | 0 | 0 | Sackville | NB |  |  |
| 2016 | C052 | Shrew | 0 | 0 | Sackville | NB |  |  |
| 2016 | C053 | Meadow vole | 0 | 0 | Sackville | NB |  |  |
| 2016 | C054 | Meadow vole | 0 | 0 | Sackville | NB |  |  |
| 2016 | C055 | Meadow vole | 0 | 0 | Sackville | NB |  |  |
| 2016 | C056 | Meadow vole | 0 | 0 | Aulac | NB |  |  |
| 2016 | C057 | Shrew | 0 | 0 | Aulac | NB |  |  |
| 2016 | C058 | Shrew | 0 | 0 | Sackville | NB |  |  |
| 2016 | C059 | Meadow vole | 0 | 0 | Sackville | NB |  |  |
| 2016 | C060 | Meadow vole | 0 | 0 | Sackville | NB |  |  |
| 2016 | C061 | Meadow vole | 0 | 0 | Sackville | NB |  |  |
| 2016 | C062 | Meadow vole | 0 | 0 | Sackville | NB |  |  |
| 2016 | C063 | Belted kingfisher | 0 | 0 | Sackville | NB |  |  |
| 2016 | C064 | Meadow vole | 0 | 0 | Sackville | NB |  |  |
| 2016 | C065 | Brown rat | 0 | 0 | Jolicure | NB |  |  |
| 2016 | C066 | Meadow vole | 0 | 0 | Jolicure | NB |  |  |
| 2016 | C067 | Meadow vole | 0 | 0 | Jolicure | NB |  |  |
| 2016 | C068 | Meadow vole | 0 | 0 | Jolicure | NB |  |  |
| 2016 | C069 | Meadow vole | 0 | 0 | Jolicure | NB |  |  |
| 2016 | C070 | Meadow vole | 0 | 0 | Jolicure | NB |  |  |
| 2016 | C071 | Meadow vole | 0 | 0 | Jolicure | NB |  |  |
| 2016 | C072 | Meadow vole | 0 | 0 | Jolicure | NB |  |  |
| 2016 | C073 | Meadow vole | 0 | 0 | Jolicure | NB |  |  |
| 2016 | C074 | Meadow vole | 0 | 0 | Jolicure | NB |  |  |
| 2016 | C075 | Meadow vole | 0 | 0 | Tidnish | NS |  |  |
| 2016 | C076 | Meadow vole | 0 | 0 | Tidnish | NS |  |  |
| 2016 | C077 | Meadow vole | 0 | 0 | Tidnish | NS |  |  |
| 2016 | C078 | Meadow vole | 0 | 0 | Tidnish | NS |  |  |
| 2016 | C079 | Shrew | 0 | 0 | Tidnish | NS |  |  |
| 2016 | C080 | Meadow vole | 0 | 0 | Sackville | NB |  |  |
| 2016 | C081 | Meadow vole | 0 | 0 | Sackville | NB |  |  |
| 2016 | C082 | Shrew | 0 | 0 | Sackville | NB |  |  |
| 2016 | C083 | Unknown | 0 | 0 | Sackville | NB |  |  |
| 2016 | C084 | Unknown | 0 | 0 | Sackville | NB |  |  |
| 2016 | C085 | Unknown | 0 | 0 | Sackville | NB |  |  |
| 2016 | C086 | Unknown | 0 | 0 | Sackville | NB |  |  |
| 2016 | C087 | Unknown | 0 | 0 | Sackville | NB |  |  |
| 2016 | C088 | Unknown | 0 | 0 | Sackville | NB |  |  |
| 2016 | C089 | Unknown | 0 | 0 | Sackville | NB |  |  |
| 2016 | C090 | Unknown | 0 | 0 | Sackville | NB |  |  |
| 2016 | C091 | Meadow vole | 0 | 0 | Sackville | NB |  |  |
| 2016 | C092 | Meadow vole | 0 | 0 | Sackville | NB |  |  |
| 2016 | C093 | Meadow vole | 0 | 0 | Sackville | NB |  |  |
| 2016 | C094 | Meadow vole | 0 | 0 | Sackville | NB |  |  |
| 2016 | C095 | Meadow vole | 0 | 0 | Sackville | NB |  |  |
| 2016 | C096 | Meadow vole | 0 | 0 | Sackville | NB |  |  |
| 2016 | C097 | Meadow vole | 0 | 0 | Sackville | NB |  |  |
| 2016 | C098 | Unknown | 0 | 0 | Sackville | NB |  |  |
| 2016 | C099 | Meadow vole | 0 | 0 | Sackville | NB |  |  |
| 2016 | C100 | Meadow vole | 0 | 0 | Sackville | NB |  |  |
| 2016 | C101 | Meadow vole | 0 | 0 | Sackville | NB |  |  |
| 2016 | C102 | Meadow vole | 0 | 0 | Sackville | NB |  |  |
| 2016 | C103 | Meadow vole | 0 | 0 | Sackville | NB |  |  |
| 2016 | C104 | Meadow vole | 0 | 0 | Sackville | NB |  |  |
| 2016 | C105 | Meadow vole | 0 | 0 | Sackville | NB |  |  |
| 2016 | C106 | Meadow vole | 0 | 0 | Sackville | NB |  |  |
| 2016 | C107 | Meadow vole | 0 | 0 | Sackville | NB |  |  |
| 2016 | C108 | Meadow vole | 0 | 0 | Sackville | NB |  |  |
| 2016 | C109 | Meadow vole | 0 | 0 | Sackville | NB |  |  |
| 2016 | C110 | Deer mouse | 0 | 0 | Sackville | NB |  |  |
| 2016 | C111 | Meadow vole | 0 | 0 | Sackville | NB |  |  |
| 2016 | C112 | Meadow vole | 0 | 0 | Sackville | NB |  |  |
| 2016 | C113 | Common redpoll | 0 | 0 | Sackville | NB |  |  |
| 2016 | C114 | Common redpoll | 0 | 0 | Sackville | NB |  |  |
| 2016 | C115 | Deer mouse | 0 | 0 | Tidnish | NS |  |  |
| 2016 | C116 | Meadow vole | 0 | 0 | Tidnish | NS |  |  |
| 2016 | C117 | Meadow vole | 0 | 0 | Sackville | NB |  |  |
| 2016 | C118 | Jumping mouse | 0 | 0 | Tidnish | NS |  |  |
| 2016 | C119 | Meadow vole | 0 | 0 | Tidnish | NS |  |  |
| 2016 | C120 | Meadow vole | 0 | 0 | Tidnish | NS |  |  |
| 2016 | C121 | Deer mouse | 0 | 0 | Cumberland county | NS |  |  |
| 2016 | C122 | Meadow vole | 0 | 0 | Sackville | NB |  |  |
| 2016 | C123 | Shrew | 0 | 0 | Tidnish | NS |  |  |
| 2016 | C124 | Jumping mouse | 0 | 0 | Aulac | NB |  |  |
| 2016 | C125 | Jumping mouse | 0 | 0 | Aulac | NB |  |  |
| 2016 | C126 | Meadow vole | 0 | 0 | Aulac | NB |  |  |
| 2016 | C127 | Meadow vole | 0 | 0 | Aulac | NB |  |  |
| 2016 | C128 | Pine siskin | 0 | 0 | Millidgeville | NB |  |  |
| 2016 | C129 | American goldfinch | 0 | 0 | Sackville | NB |  |  |
| 2016 | C130 | Meadow vole | 0 | 0 | Sackville | NB |  |  |
| 2016 | C131 | Common redpoll | 0 | 0 | Sackville | NB |  |  |
| 2016 | C132 | Meadow vole | 0 | 0 | Sackville | NB |  |  |
| 2016 | C133 | Meadow vole | 0 | 0 | Sackville | NB |  |  |
| 2016 | C134 | Meadow vole | 0 | 0 | Sackville | NB |  |  |
| 2016 | C135 | Unknown | 0 | 0 | Sackville | NB |  |  |
| 2016 | C136 | Unknown | 0 | 0 | Sackville | NB |  |  |
| 2016 | C137 | Unknown | 0 | 0 | Sackville | NB |  |  |
| 2016 | C138 | Meadow vole | 0 | 0 | Tidnish | NS |  |  |
| 2016 | C139 | Pine siskin | 0 | 0 | Sackville | NB |  |  |
| 2016 | C140 | Meadow vole | 0 | 0 | Sackville | NB |  |  |
| 2016 | C141 | Meadow vole | 0 | 0 | Sackville | NB |  |  |
| 2016 | C142 | Meadow vole | 0 | 0 | Sackville | NB |  |  |
| 2016 | C143 | Meadow vole | 1 | 0 | Sackville | NB |  |  |
| 2016 | C144 | Meadow vole | 1 | 0 | Sackville | NB |  |  |
| 2016 | C145 | Meadow vole | 0 | 0 | Sackville | NB |  |  |
| 2016 | C146 | Meadow vole | 0 | 0 | Sackville | NB |  |  |
| 2016 | C147 | Meadow vole | 1 | 0 | Sackville | NB |  |  |
| 2016 | C148 | Meadow vole | 0 | 0 | Sackville | NB |  |  |
| 2016 | C149 | Meadow vole | 0 | 0 | Jolicure | NB |  |  |
| 2016 | C150 | Meadow vole | 0 | 0 | Jolicure | NB |  |  |
| 2016 | C151 | Meadow vole | 0 | 0 | Jolicure | NB |  |  |
| 2016 | C152 | Meadow vole | 0 | 0 | Jolicure | NB |  |  |
| 2016 | C153 | Meadow vole | 0 | 0 | Jolicure | NB |  |  |
| 2016 | C154 | Meadow vole | 0 | 0 | Jolicure | NB |  |  |
| 2016 | C155 | Deer mouse | 0 | 0 | Jolicure | NB |  |  |
| 2016 | C156 | Meadow vole | 0 | 0 | Jolicure | NB |  |  |
| 2016 | C157 | Deer mouse | 0 | 0 | Jolicure | NB |  |  |
| 2016 | C158 | Meadow vole | 0 | 0 | Jolicure | NB |  |  |
| 2016 | C159 | Jumping mouse | 0 | 0 | Jolicure | NB |  |  |
| 2016 | C160 | Meadow vole | 0 | 0 | Jolicure | NB |  |  |
| 2016 | C161 | Meadow vole | 0 | 0 | Jolicure | NB |  |  |
| 2016 | C162 | Meadow vole | 0 | 0 | Jolicure | NB |  |  |
| 2016 | C163 | Unknown | 0 | 0 | Jolicure | NB |  |  |
| 2016 | C164 | Meadow vole | 0 | 0 | Jolicure | NB |  |  |
| 2016 | C165 | Meadow vole | 0 | 1 | Jolicure | NB |  |  |
| 2016 | C166 | Shrew | 0 | 0 | Jolicure | NB |  |  |
| 2016 | C167 | Meadow vole | 0 | 0 | Jolicure | NB |  |  |
| 2016 | C168 | Meadow vole | 0 | 0 | Aulac | NB |  |  |
| 2016 | C169 | Meadow vole | 0 | 0 | Aulac | NB |  |  |
| 2016 | C170 | Meadow vole | 0 | 0 | Aulac | NB |  |  |
| 2016 | C171 | Meadow vole | 0 | 0 | Aulac | NB |  |  |
| 2016 | C172 | Meadow vole | 0 | 0 | Aulac | NB |  |  |
| 2016 | C173 | Meadow vole | 0 | 0 | Aulac | NB |  |  |
| 2016 | C174 | Meadow vole | 0 | 0 | Jolicure | NB |  |  |
| 2016 | C175 | Deer mouse | 0 | 0 | Jolicure | NB |  |  |
| 2016 | C176 | Deer mouse | 0 | 0 | Jolicure | NB |  |  |
| 2016 | C177 | Deer mouse | 0 | 0 | Jolicure | NB |  |  |
| 2016 | C178 | Meadow vole | 0 | 0 | Jolicure | NB |  |  |
| 2016 | C179 | Meadow vole | 0 | 0 | Jolicure | NB |  |  |
| 2016 | C180 | Meadow vole | 0 | 0 | Jolicure | NB |  |  |
| 2016 | C181 | Meadow vole | 0 | 0 | Jolicure | NB |  |  |
| 2016 | C182 | Meadow vole | 0 | 0 | Sackville | NB |  |  |
| 2016 | C183 | Meadow vole | 0 | 0 | Sackville | NB |  |  |
| 2016 | C184 | Meadow vole | 0 | 0 | Sackville | NB |  |  |
| 2016 | C185 | Meadow vole | 0 | 0 | Sackville | NB |  |  |
| 2016 | C186 | Meadow vole | 0 | 0 | Sackville | NB |  |  |
| 2016 | C187 | Meadow vole | 0 | 0 | Sackville | NB |  |  |
| 2016 | C188 | Meadow vole | 0 | 0 | Sackville | NB |  |  |
| 2016 | C189 | Meadow vole | 0 | 0 | Sackville | NB |  |  |
| 2016 | C190 | Meadow vole | 0 | 0 | Sackville | NB |  |  |
| 2016 | C191 | Meadow vole | 0 | 0 | Sackville | NB |  |  |
| 2016 | C192 | Meadow vole | 0 | 0 | Sackville | NB |  |  |
| 2016 | C193 | Brown rat | 0 | 0 | Sackville | NB |  |  |
| 2016 | C194 | House finch | 0 | 0 | Sackville | NB |  |  |
| 2016 | C195 | Deer mouse | 0 | 0 | Cumberland county | NS |  |  |
| 2016 | C196 | Unknown | 0 | 0 | Cumberland county | NS |  |  |
| 2016 | C197 | Jumping mouse | 0 | 0 | Cumberland county | NS |  |  |
| 2016 | C198 | Meadow vole | 0 | 0 | Cumberland county | NS |  |  |
| 2016 | C199 | Deer mouse | 0 | 0 | Cumberland county | NS |  |  |
| 2016 | C200 | Meadow vole | 0 | 0 | Cumberland county | NS |  |  |
| 2016 | C201 | Meadow vole | 0 | 0 | Cumberland county | NS |  |  |
| 2016 | C202 | Meadow vole | 0 | 0 | Cumberland county | NS |  |  |
| 2016 | C203 | Deer mouse | 1 | 0 | Cumberland county | NS |  |  |
| 2016 | C204 | Deer mouse | 1 | 0 | Cumberland county | NS |  |  |
| 2016 | C205 | Deer mouse | 0 | 0 | Cumberland county | NS |  |  |
| 2016 | C206 | Shrew | 1 | 0 | Sackville | NB |  |  |
| 2016 | C207 | Shrew | 0 | 0 | Sackville | NB |  |  |
| 2017 | C001 | Shrew | 0 | 0 | Aulac | NB |  |  |
| 2017 | C002 | Snow shoe hare | 0 | 0 | St. Martins | NB |  |  |
| 2017 | C003 | Jumping mouse | 0 | 0 | Sackville | NB |  |  |
| 2017 | C004 | Jumping mouse | 0 | 0 | Sackville | NB |  |  |
| 2017 | C005 | Shrew | 0 | 0 | Sackville | NB |  |  |
| 2017 | C006 | Jumping mouse | 0 | 0 | Sackville | NB |  |  |
| 2017 | C007 | Meadow vole | 0 | 0 | Aulac | NB |  |  |
| 2017 | C008 | Deer mouse | 0 | 0 | Aulac | NB |  |  |
| 2017 | C009 | Downy woodpecker | 0 | 0 | Tidnish | NS |  |  |
| 2017 | C010 | Meadow vole | 0 | 0 | Tidnish | NS |  |  |
| 2017 | C011 | Meadow vole | 0 | 0 | Tidnish | NS |  |  |
| 2017 | C012 | Brown rat | 0 | 0 | Jolicure | NB |  |  |
| 2017 | C013 | Jumping mouse | 0 | 0 | Jolicure | NB |  |  |
| 2017 | C014 | Jumping mouse | 0 | 0 | Jolicure | NB |  |  |
| 2017 | C015 | Jumping mouse | 0 | 0 | Sackville | NB |  |  |
| 2017 | C016 | Brown rat | 0 | 0 | Jolicure | NB |  |  |
| 2017 | C017 | Short tailed weasel | 0 | 0 | Jolicure | NB |  |  |
| 2017 | C018 | Meadow vole | 0 | 0 | Sackville | NB |  |  |
| 2017 | C019 | Meadow vole | 0 | 0 | Jolicure | NB |  |  |
| 2017 | C020 | Meadow vole | 0 | 0 | Jolicure | NB |  |  |
| 2017 | C021 | Meadow vole | 0 | 0 | Jolicure | NB |  |  |
| 2017 | C022 | Meadow vole | 0 | 0 | Jolicure | NB |  |  |
| 2017 | C023 | Meadow vole | 0 | 0 | Jolicure | NB |  |  |
| 2017 | C024 | Eastern grey squirrel | 0 | 0 | Fredericton | NB |  |  |
| 2017 | C025 | Unknown | 0 | 0 | Aulac | NB |  |  |
| 2017 | C026 | Ruby-throated hummingbird | 0 | 0 | Aulac | NB |  |  |
| 2017 | C027 | Meadow vole | 0 | 0 | Sackville | NB |  |  |
| 2017 | C028 | Meadow vole | 0 | 0 | Sackville | NB |  |  |
| 2017 | C029 | Meadow vole | 0 | 0 | Sackville | NB |  |  |
| 2017 | C030 | Meadow vole | 0 | 0 | Sackville | NB |  |  |
| 2017 | C031 | Meadow vole | 0 | 0 | Sackville | NB |  |  |
| 2017 | C032 | Meadow vole | 0 | 0 | Sackville | NB |  |  |
| 2017 | C033 | Shrew | 0 | 0 | Sackville | NB |  |  |
| 2017 | C034 | Shrew | 0 | 0 | Sackville | NB |  |  |
| 2017 | C035 | Shrew | 0 | 0 | Aulac | NB |  |  |
| 2017 | C036 | Shrew | 0 | 0 | Aulac | NB |  |  |
| 2017 | C037 | Jumping mouse | 0 | 0 | Aulac | NB |  |  |
| 2017 | C038 | Meadow vole | 0 | 0 | Sackville | NB |  |  |
| 2017 | C039 | Shrew | 0 | 0 | Sackville | NB |  |  |
| 2017 | C040 | Chipmunk | 0 | 0 | Burton | NB |  |  |
| 2017 | C041 | Eastern grey squirrel | 0 | 0 | Fredericton | NB |  |  |
| 2017 | C042 | Raccoon | 0 | 0 | Fredericton | NB |  |  |
| 2017 | C043 | Unknown | 0 | 0 | Sackville | NB |  |  |
| 2017 | C044 | Shrew | 0 | 0 | Sackville | NB |  |  |
| 2017 | C045 | Shrew | 0 | 0 | Sackville | NB |  |  |
| 2017 | C046 | Groundhog | 0 | 0 | Fredericton | NB |  |  |
| 2017 | C047 | Jumping mouse | 0 | 0 | Aulac | NB |  |  |
| 2017 | C048 | Shrew | 0 | 0 | Sackville | NB |  |  |
| 2017 | C049 | Shrew | 0 | 0 | Sackville | NB |  |  |
| 2017 | C050 | Shrew | 1 | 0 | Sackville | NB |  |  |
| 2017 | C051 | Shrew | 0 | 0 | Sackville | NB |  |  |
| 2017 | C052 | Unknown | 0 | 0 | Aulac | NB |  |  |
| 2017 | C053 | Jumping mouse | 0 | 0 | Aulac | NB |  |  |
| 2017 | C054 | Unknown | 0 | 0 | Aulac | NB |  |  |
| 2017 | C055 | Meadow vole | 0 | 0 | Aulac | NB |  |  |
| 2017 | C056 | Chipmunk | 0 | 0 | Sackville | NB |  |  |
| 2017 | C057 | Unknown | 0 | 0 | Sackville | NB |  |  |
| 2017 | C058 | Shrew | 0 | 0 | Sackville | NB |  |  |
| 2017 | C059 | Shrew | 0 | 0 | Sackville | NB |  |  |
| 2017 | C060 | Meadow vole | 0 | 0 | Aulac | NB |  |  |
| 2017 | C061 | Meadow vole | 0 | 0 | Sackville | NB |  |  |
| 2017 | C062 | Deer mouse | 0 | 1 | Sackville | NB |  |  |
| 2017 | C063 | Jumping mouse | 0 | 1 | Jolicure | NB |  |  |
| 2017 | C064 | Jumping mouse | 0 | 0 | Aulac | NB |  |  |
| 2017 | C065 | Shrew | 0 | 0 | Sackville | NB |  |  |
| 2017 | C066 | Shrew | 0 | 0 | Sackville | NB |  |  |
| 2017 | C067 | Snow shoe hare | 0 | 0 | Moncton | NB |  |  |
| 2017 | C068 | Eastern grey squirrel | 0 | 1 | Fredericton | NB |  |  |
| 2017 | C069 | Eastern grey squirrel | 0 | 0 | Fredericton | NB |  |  |
| 2017 | C070 | Unknown | 0 | 0 | Fredericton | NB |  |  |
| 2017 | C071 | Shrew | 0 | 0 | Jolicure | NB |  |  |
| 2017 | C072 | Jumping mouse | 0 | 1 | Sackville | NB |  |  |
| 2017 | C073 | Meadow vole | 0 | 0 | Aulac | NB |  |  |
| 2017 | C074 | Meadow vole | 0 | 0 | Cookville | NB |  |  |
| 2017 | C075 | Meadow vole | 0 | 0 | Moncton | NB |  |  |
